# Supplementary material for: Chromatographic Techniques and Pharmacological Analysis as a Quality Control Strategy for Serjania triquetra a Traditional Medicinal Plant
Source: Pharmaceuticals (Basel). 2022 Oct 20;15(10):1289. doi: 10.3390/ph15101289 (PMC9611020; doi:10.3390/ph15101289)
Supplement: Supplementary file 1 [file pharmaceuticals-15-01289-s001.zip › pharmaceuticals-1907281 Supplementary Material St.pptx]

## Slide 1
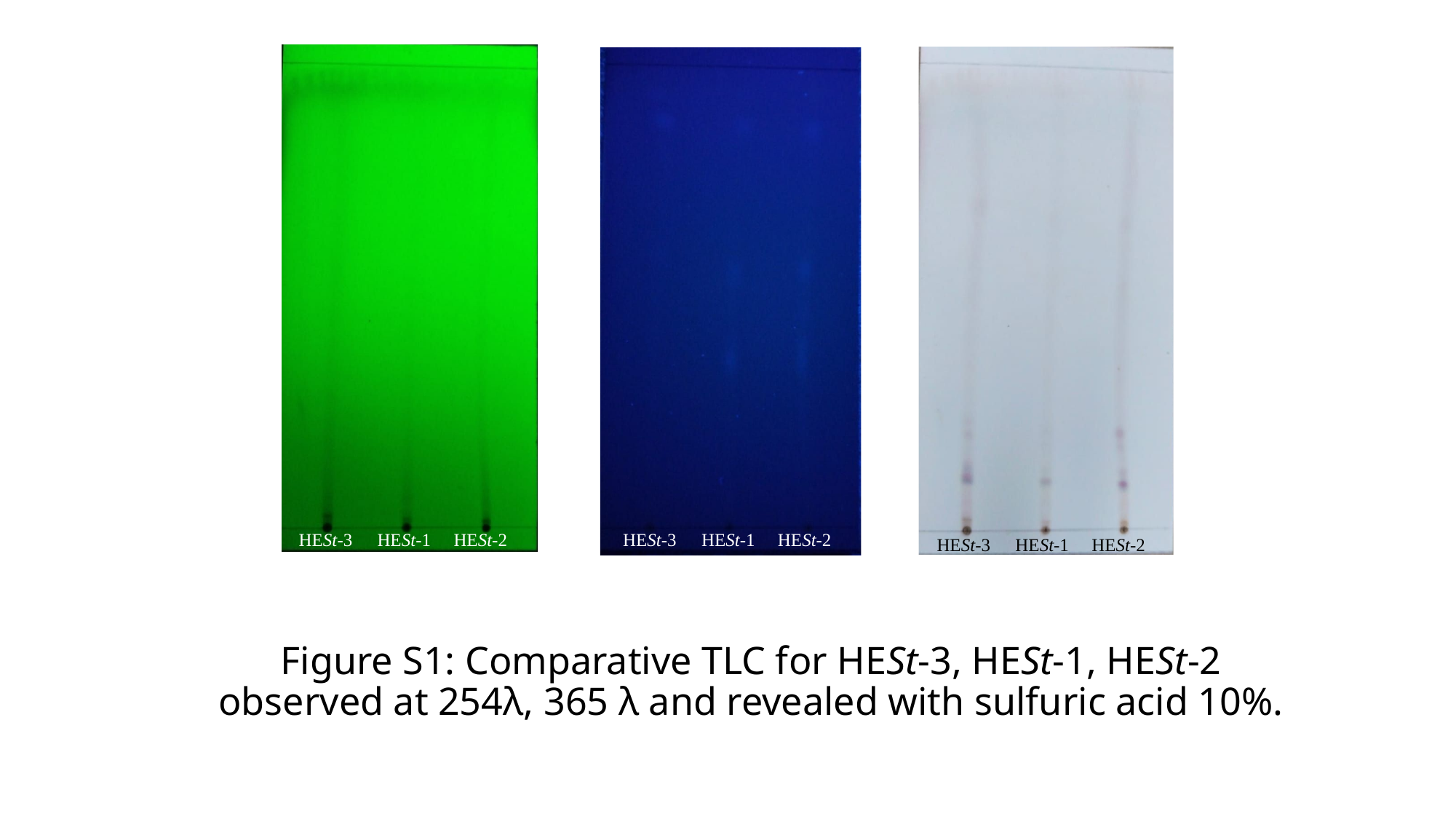

HESt-3
HESt-1
HESt-2
HESt-3
HESt-1
HESt-2
HESt-3
HESt-1
HESt-2
# Figure S1: Comparative TLC for HESt-3, HESt-1, HESt-2 observed at 254λ, 365 λ and revealed with sulfuric acid 10%.

## Slide 2
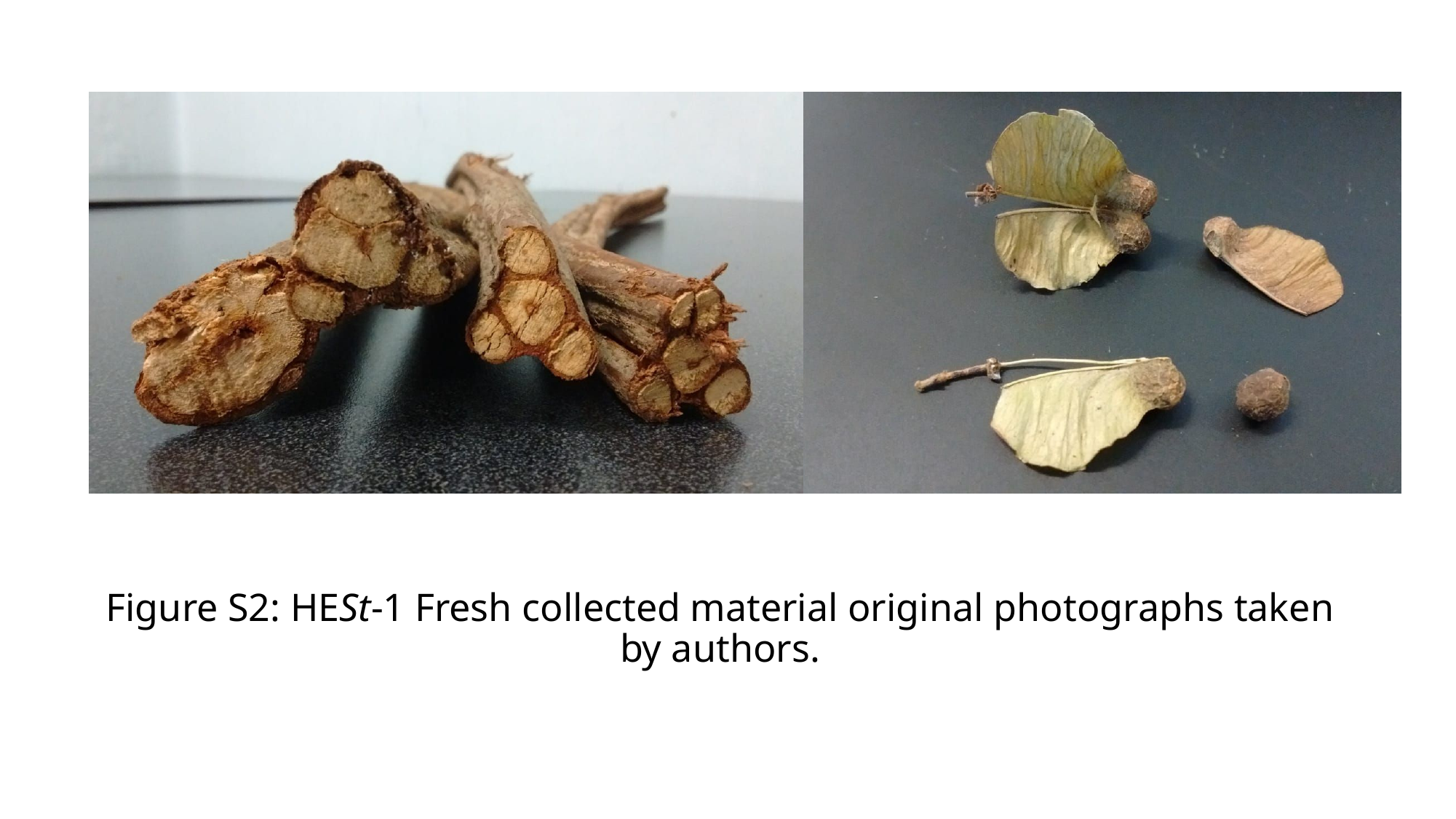

# Figure S2: HESt-1 Fresh collected material original photographs taken by authors.
Seeds and fruits from S. triquetra
Herbal material used for identification purpose only

## Slide 3
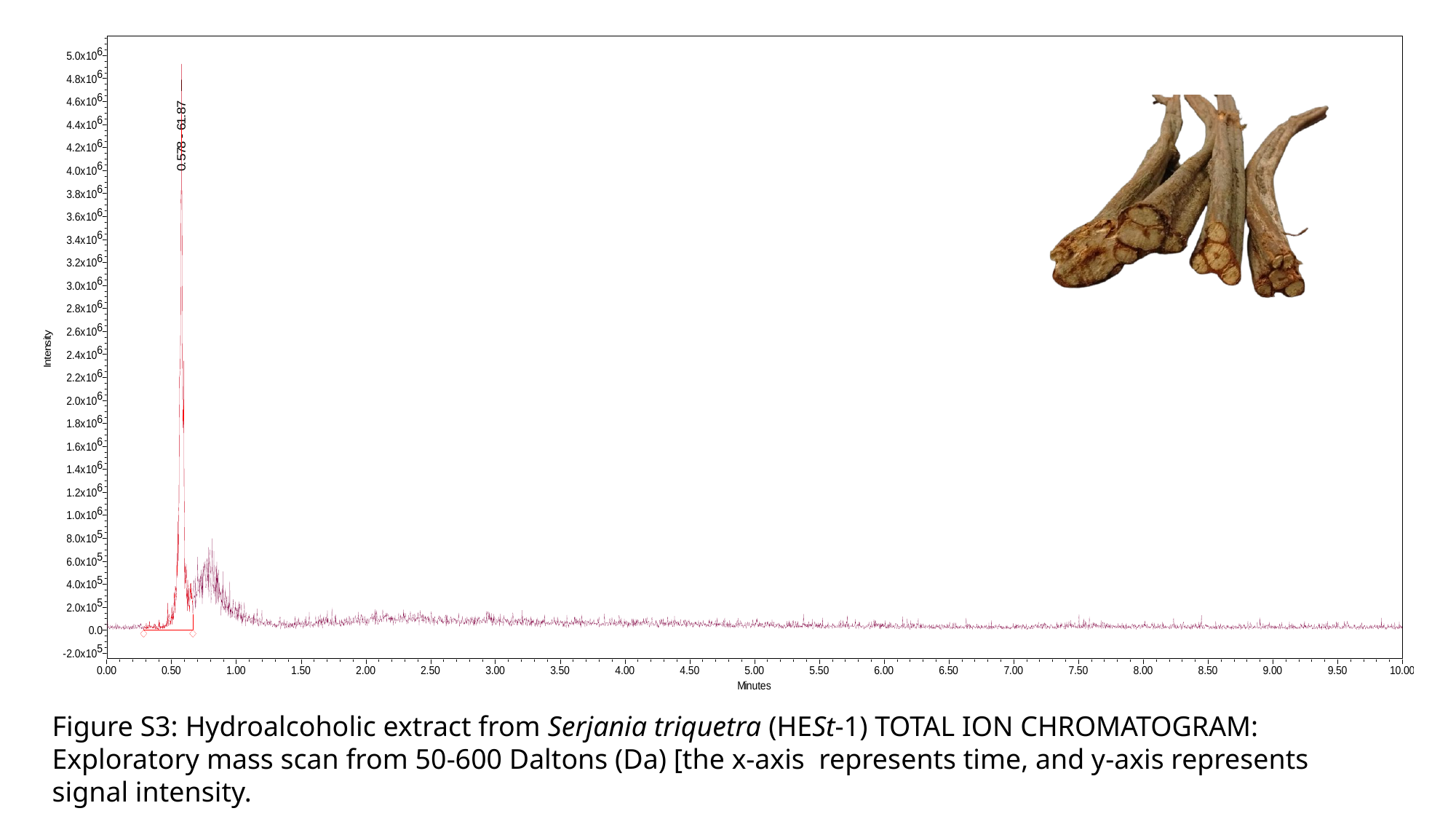

Figure S3: Hydroalcoholic extract from Serjania triquetra (HESt-1) TOTAL ION CHROMATOGRAM: Exploratory mass scan from 50-600 Daltons (Da) [the x-axis represents time, and y-axis represents signal intensity.

## Slide 4
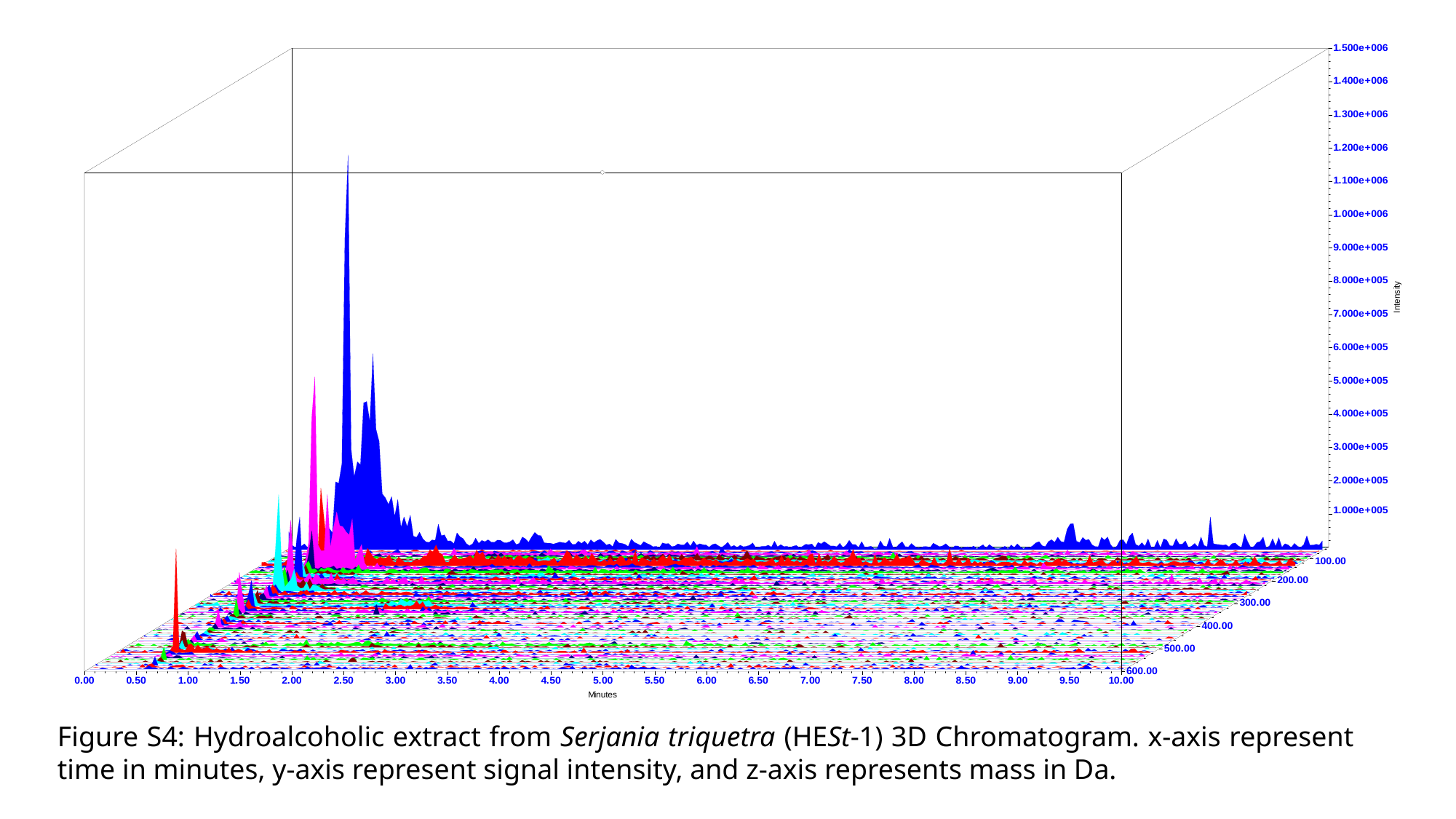

Figure S4: Hydroalcoholic extract from Serjania triquetra (HESt-1) 3D Chromatogram. x-axis represent time in minutes, y-axis represent signal intensity, and z-axis represents mass in Da.

## Slide 5
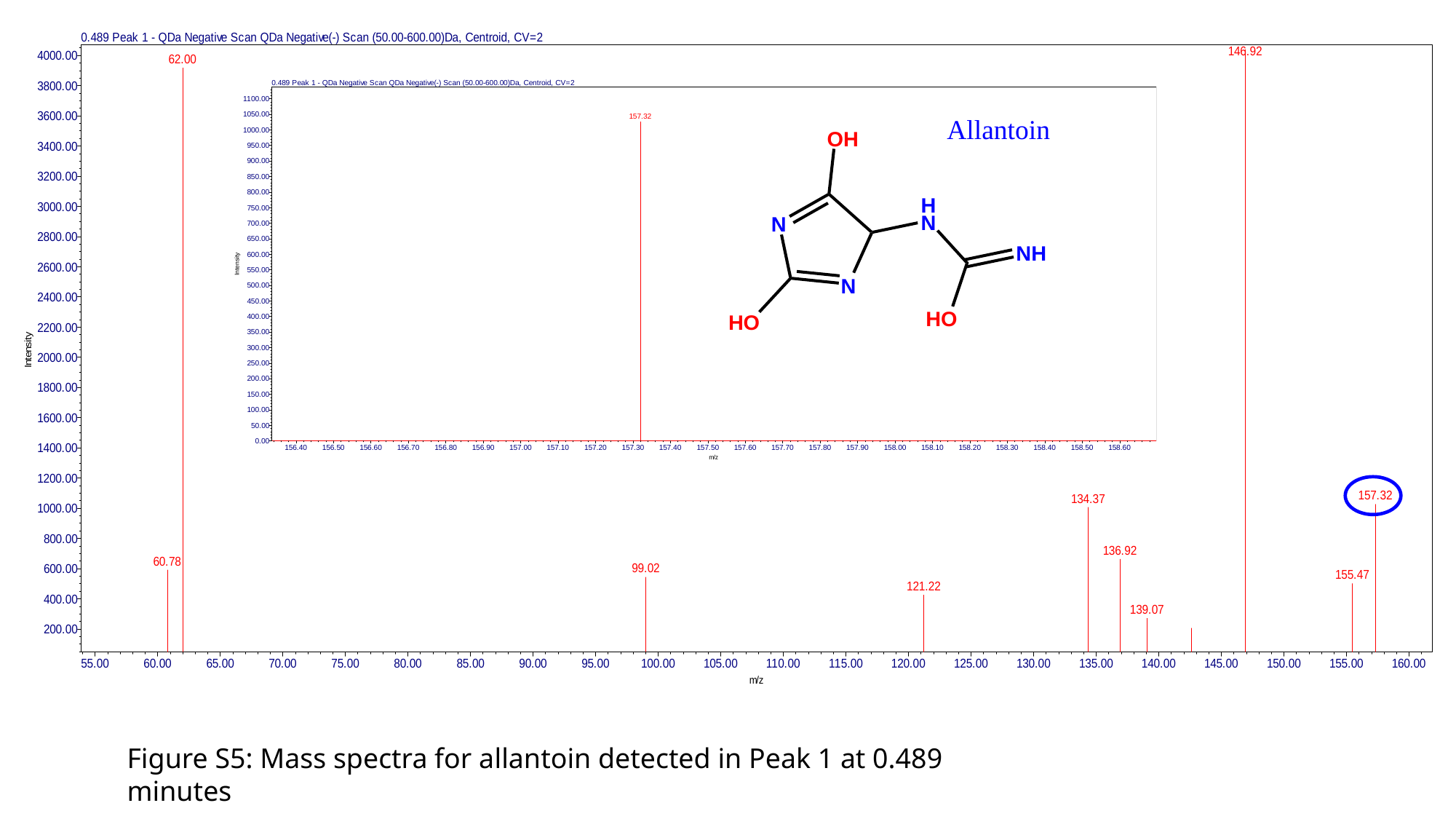

Allantoin
Figure S5: Mass spectra for allantoin detected in Peak 1 at 0.489 minutes

## Slide 6
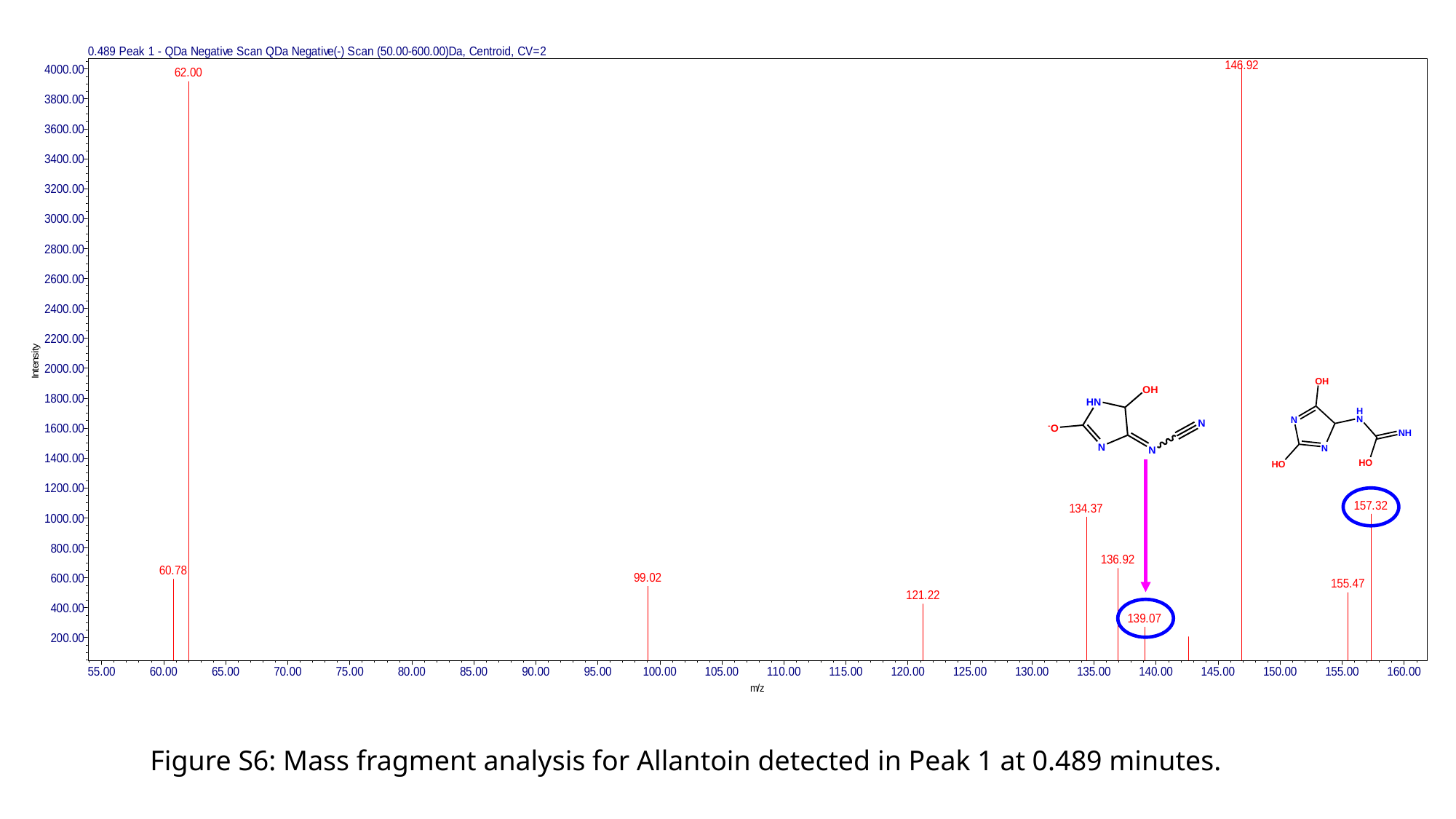

Figure S6: Mass fragment analysis for Allantoin detected in Peak 1 at 0.489 minutes.

## Slide 7
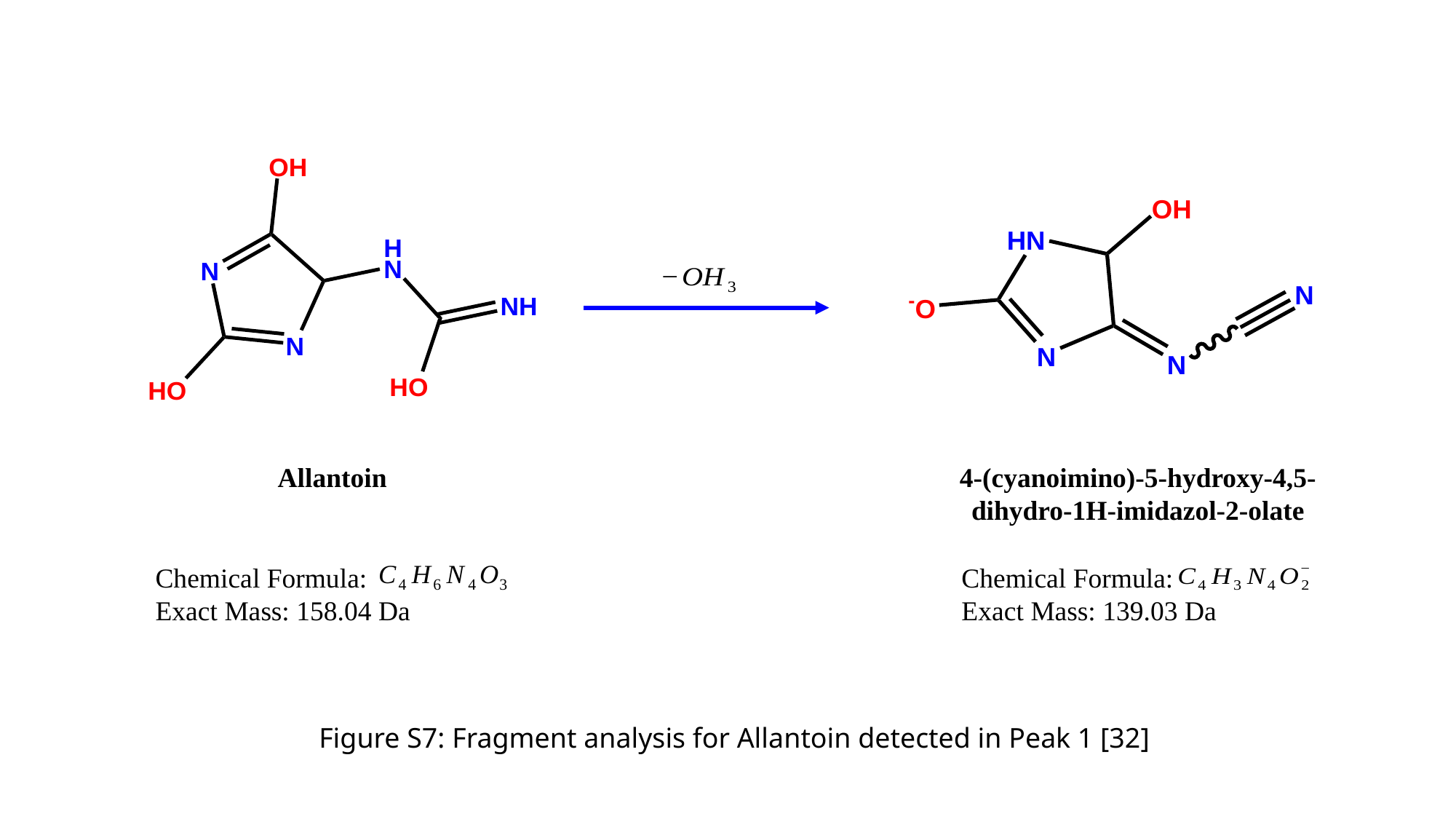

Allantoin
4-(cyanoimino)-5-hydroxy-4,5-dihydro-1H-imidazol-2-olate
Chemical Formula:
Exact Mass: 139.03 Da
Chemical Formula:
Exact Mass: 158.04 Da
Figure S7: Fragment analysis for Allantoin detected in Peak 1 [32]

## Slide 8
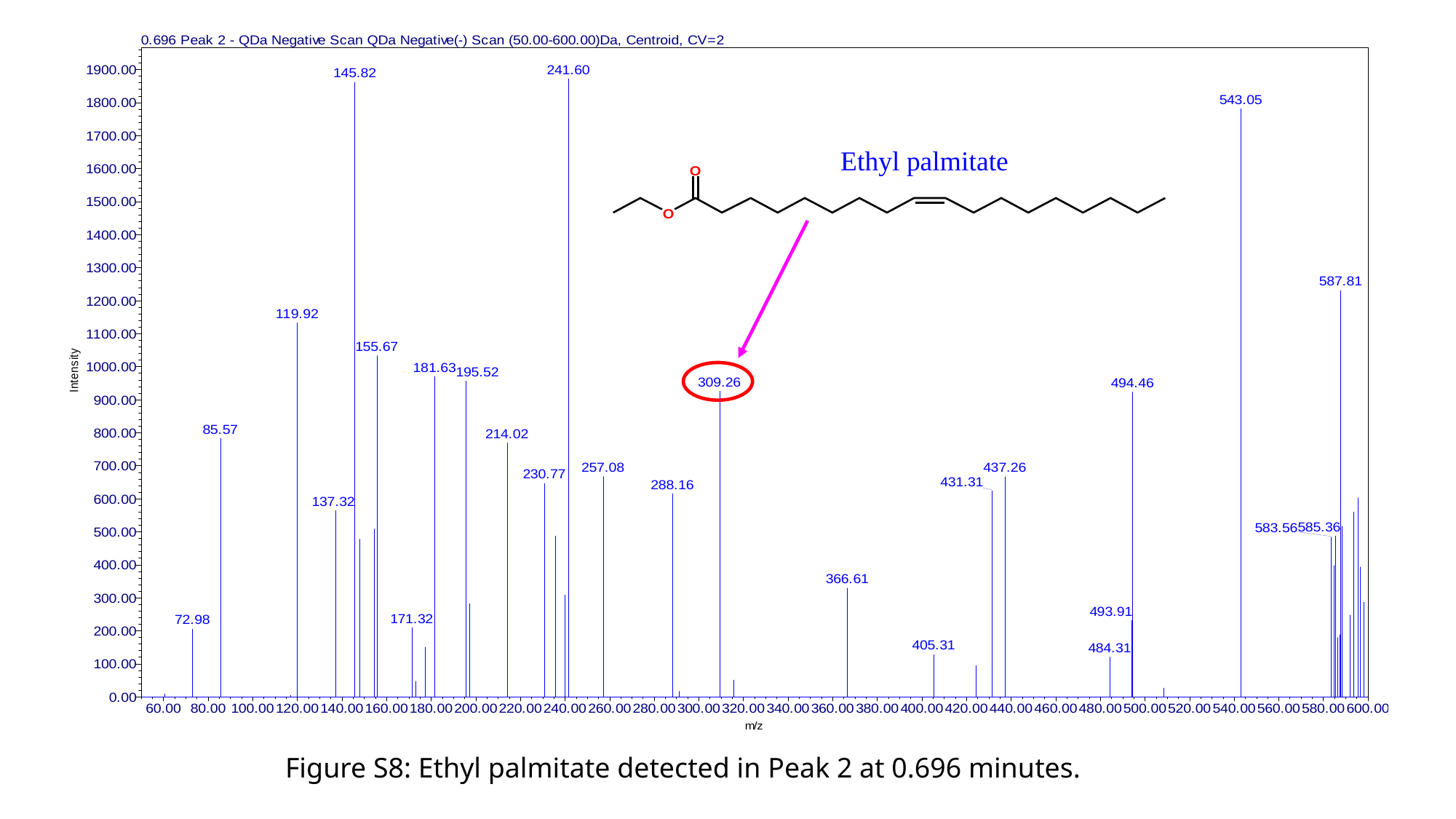

Ethyl palmitate
Figure S8: Ethyl palmitate detected in Peak 2 at 0.696 minutes.

## Slide 9
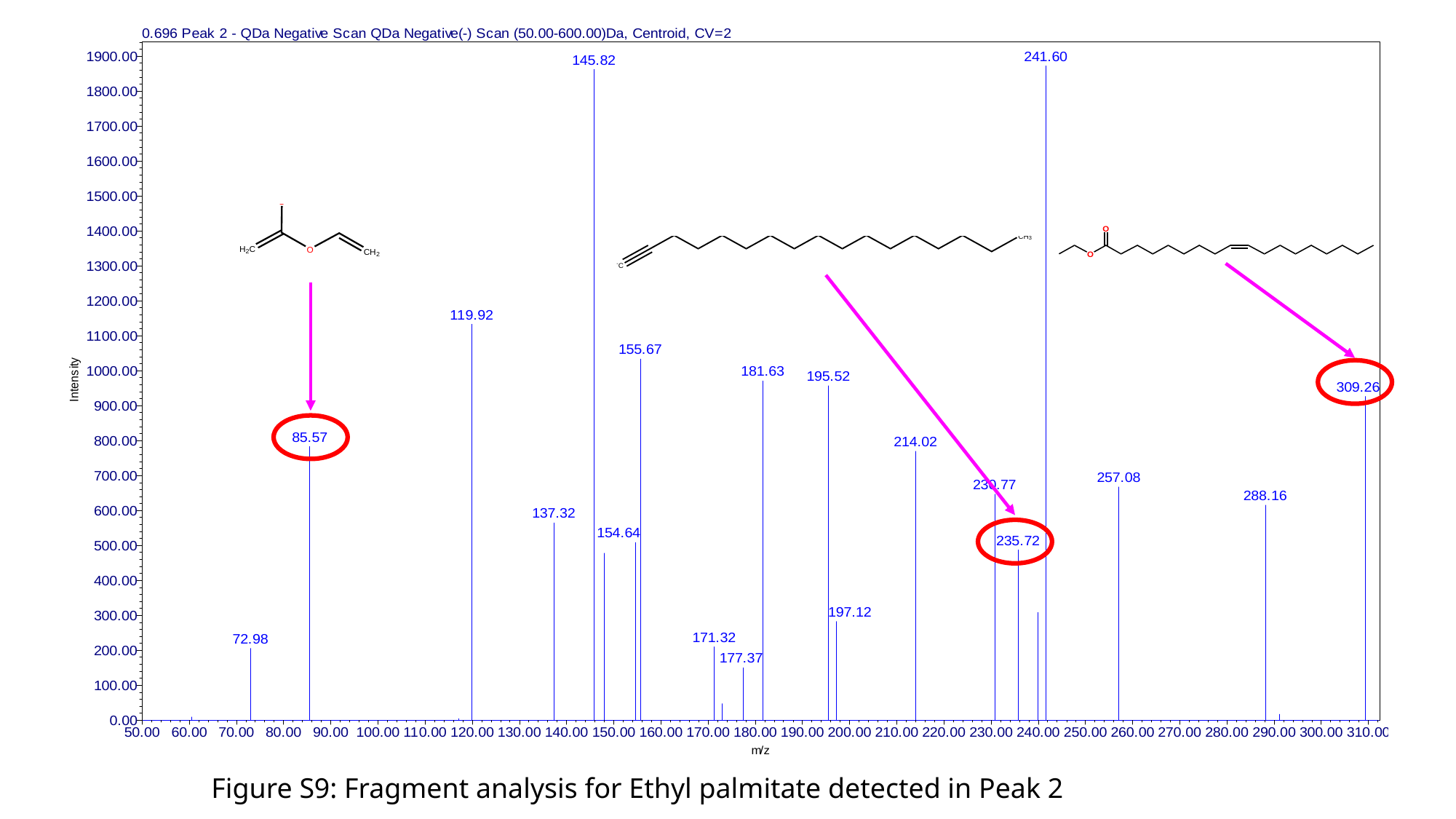

Figure S9: Fragment analysis for Ethyl palmitate detected in Peak 2

## Slide 10
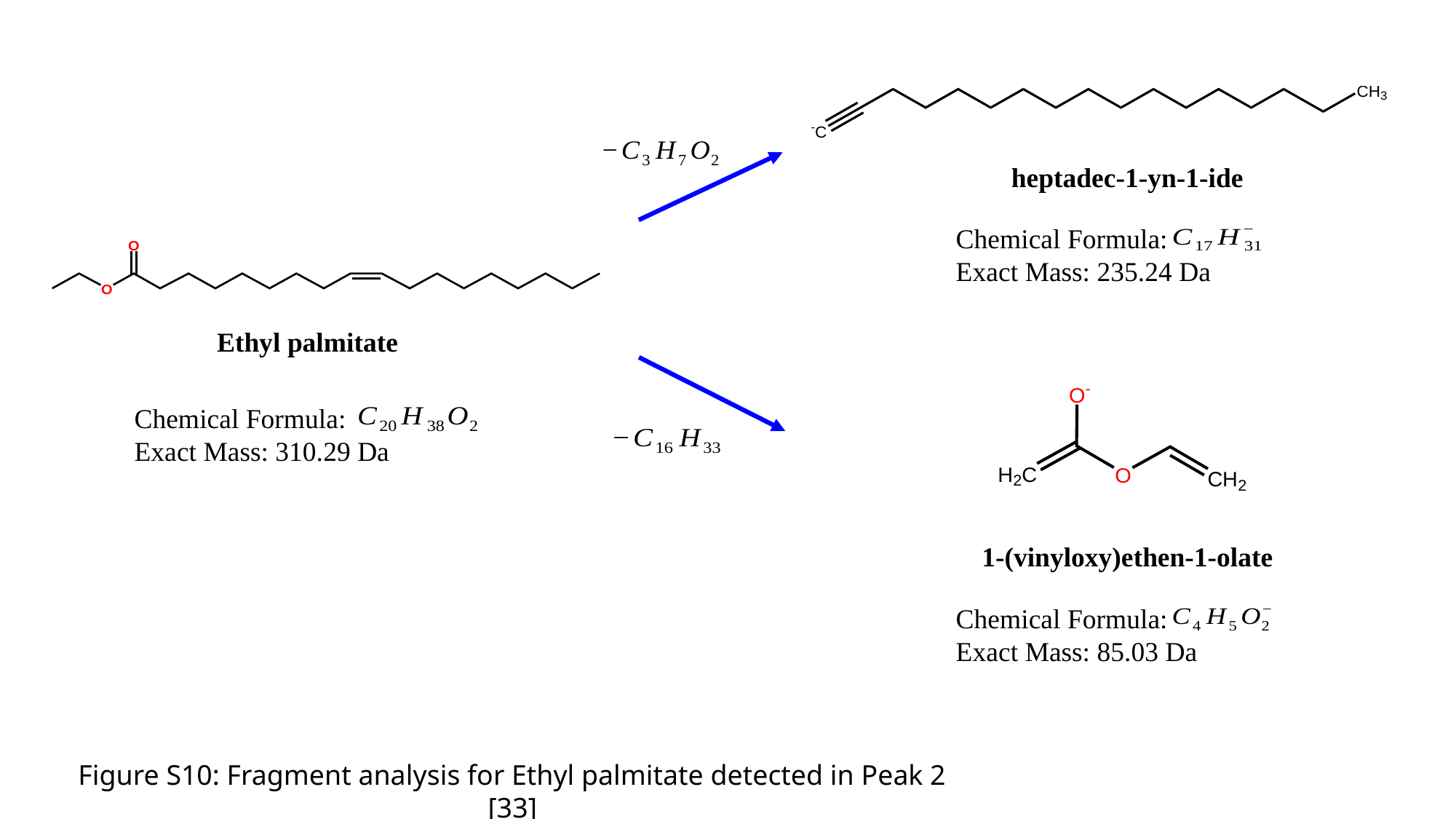

heptadec-1-yn-1-ide
Chemical Formula:
Exact Mass: 235.24 Da
Ethyl palmitate
Chemical Formula:
Exact Mass: 310.29 Da
1-(vinyloxy)ethen-1-olate
Chemical Formula:
Exact Mass: 85.03 Da
Figure S10: Fragment analysis for Ethyl palmitate detected in Peak 2 [33]

## Slide 11
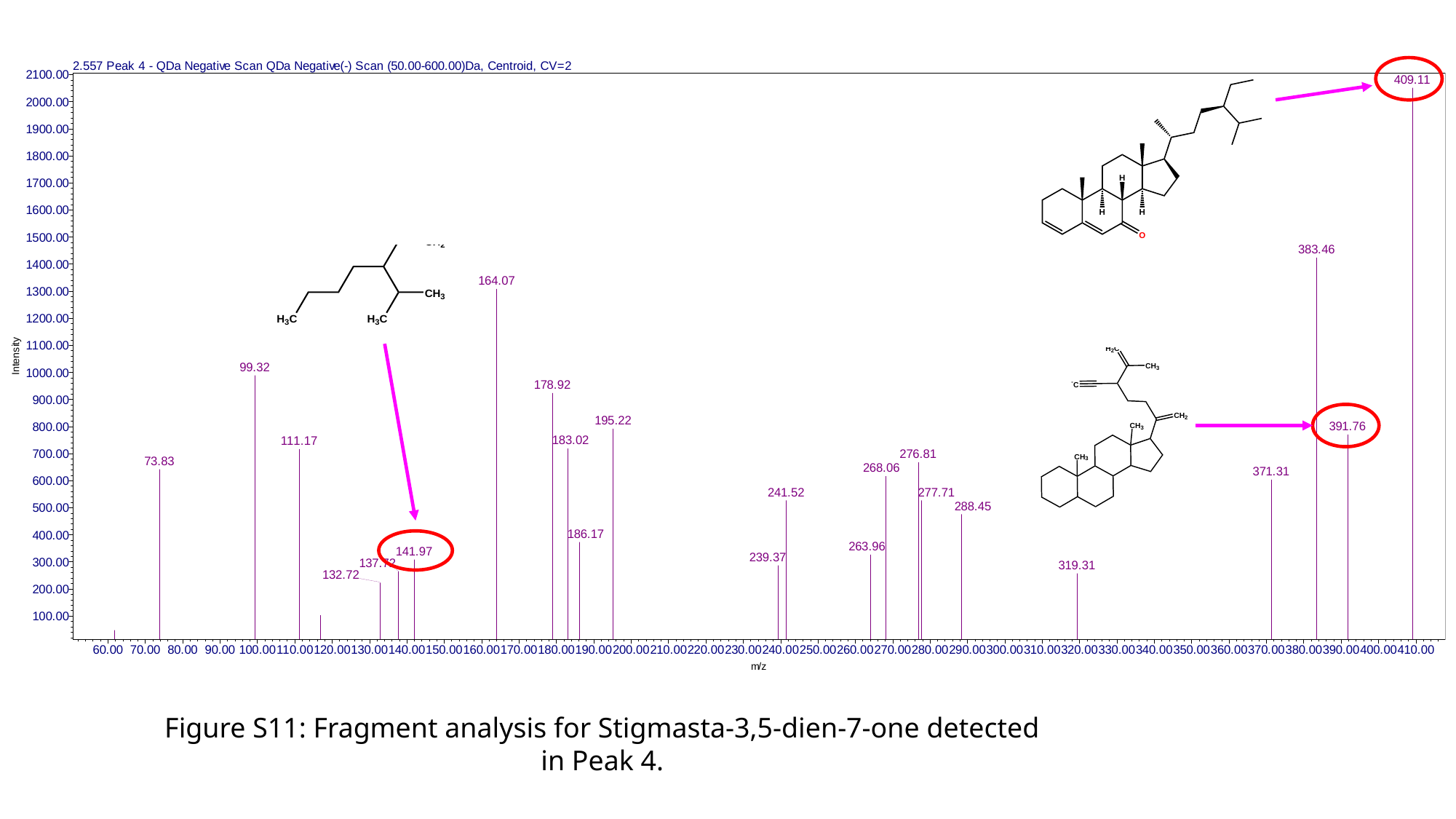

Figure S11: Fragment analysis for Stigmasta-3,5-dien-7-one detected in Peak 4.

## Slide 12
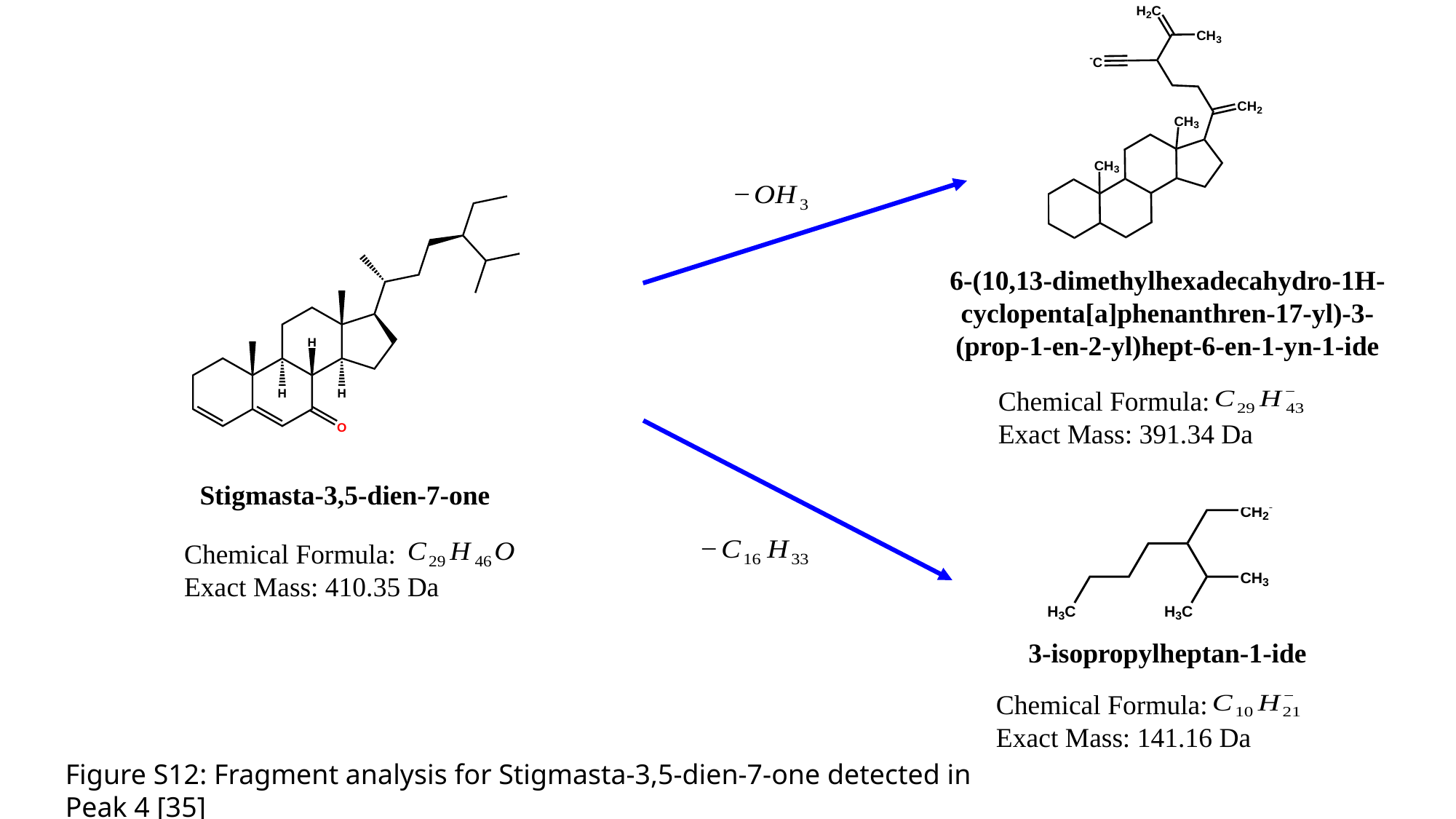

6-(10,13-dimethylhexadecahydro-1H-cyclopenta[a]phenanthren-17-yl)-3-(prop-1-en-2-yl)hept-6-en-1-yn-1-ide
Chemical Formula:
Exact Mass: 391.34 Da
Stigmasta-3,5-dien-7-one
Chemical Formula:
Exact Mass: 410.35 Da
3-isopropylheptan-1-ide
Chemical Formula:
Exact Mass: 141.16 Da
Figure S12: Fragment analysis for Stigmasta-3,5-dien-7-one detected in Peak 4 [35]

## Slide 13
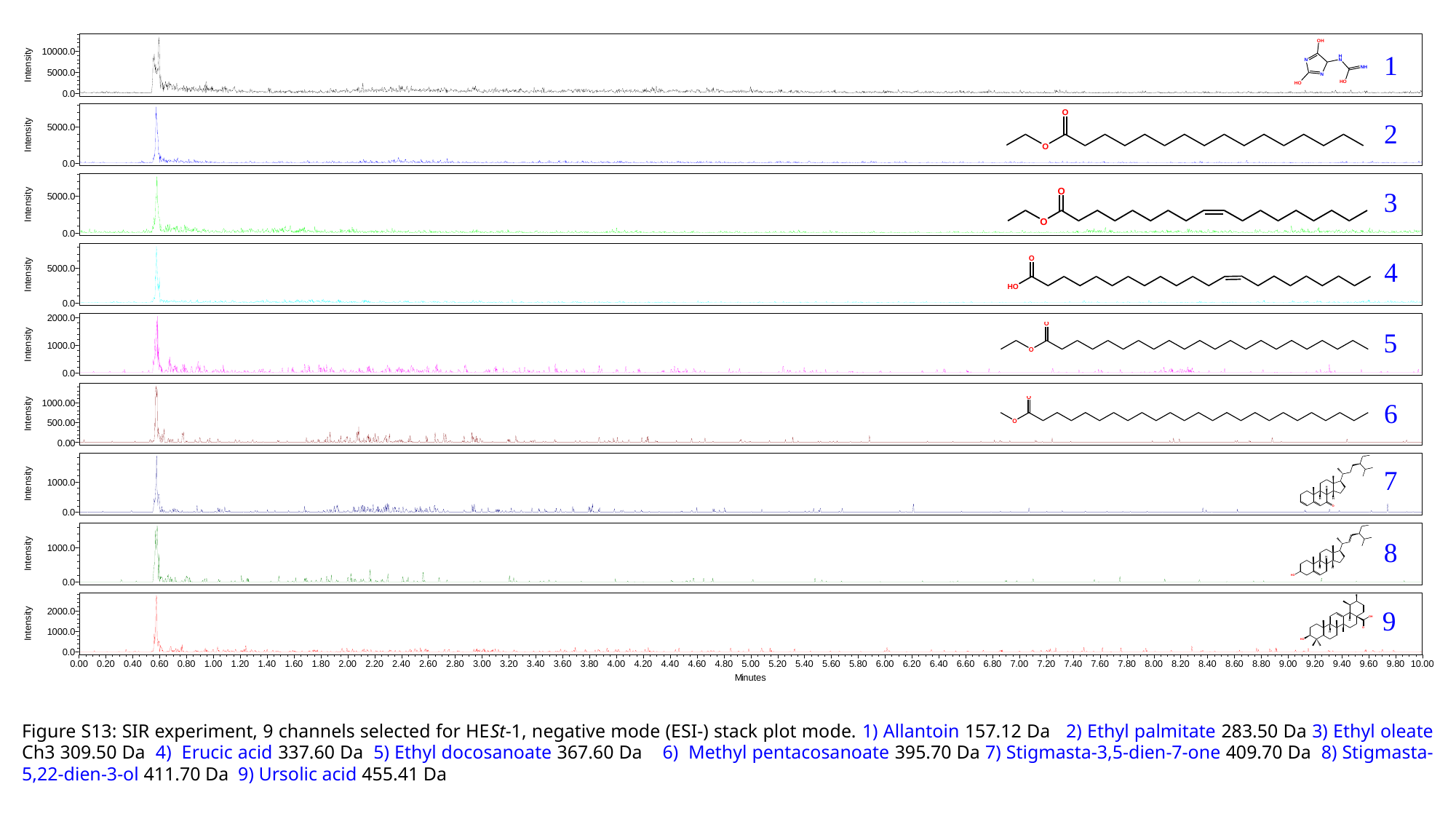

1
2
3
4
5
6
7
8
9
Figure S13: SIR experiment, 9 channels selected for HESt-1, negative mode (ESI-) stack plot mode. 1) Allantoin 157.12 Da 2) Ethyl palmitate 283.50 Da 3) Ethyl oleate Ch3 309.50 Da 4) Erucic acid 337.60 Da 5) Ethyl docosanoate 367.60 Da 6) Methyl pentacosanoate 395.70 Da 7) Stigmasta-3,5-dien-7-one 409.70 Da 8) Stigmasta-5,22-dien-3-ol 411.70 Da 9) Ursolic acid 455.41 Da

## Slide 14
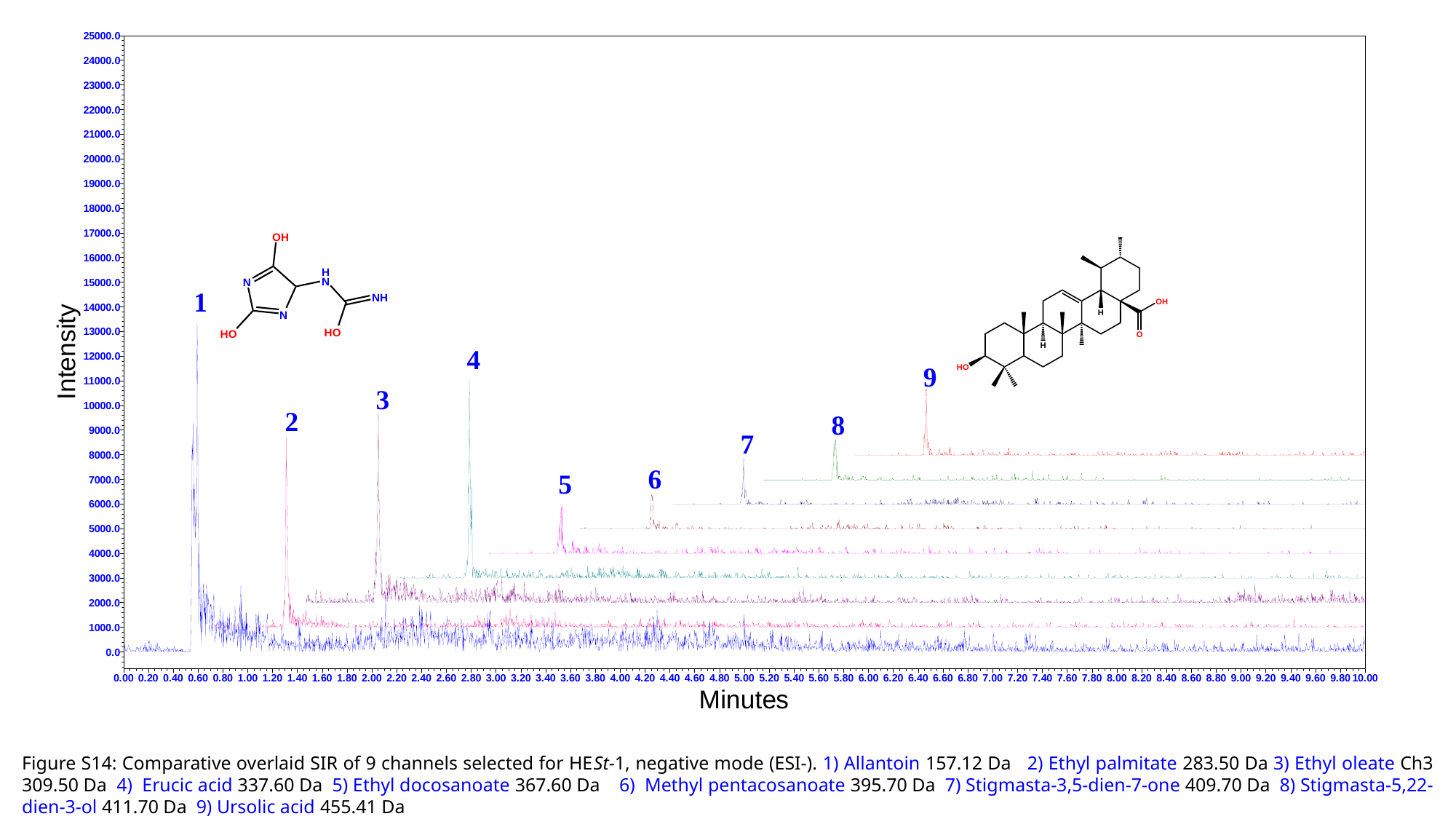

1
4
9
3
2
8
7
6
5
Figure S14: Comparative overlaid SIR of 9 channels selected for HESt-1, negative mode (ESI-). 1) Allantoin 157.12 Da 2) Ethyl palmitate 283.50 Da 3) Ethyl oleate Ch3 309.50 Da 4) Erucic acid 337.60 Da 5) Ethyl docosanoate 367.60 Da 6) Methyl pentacosanoate 395.70 Da 7) Stigmasta-3,5-dien-7-one 409.70 Da 8) Stigmasta-5,22-dien-3-ol 411.70 Da 9) Ursolic acid 455.41 Da

## Slide 15
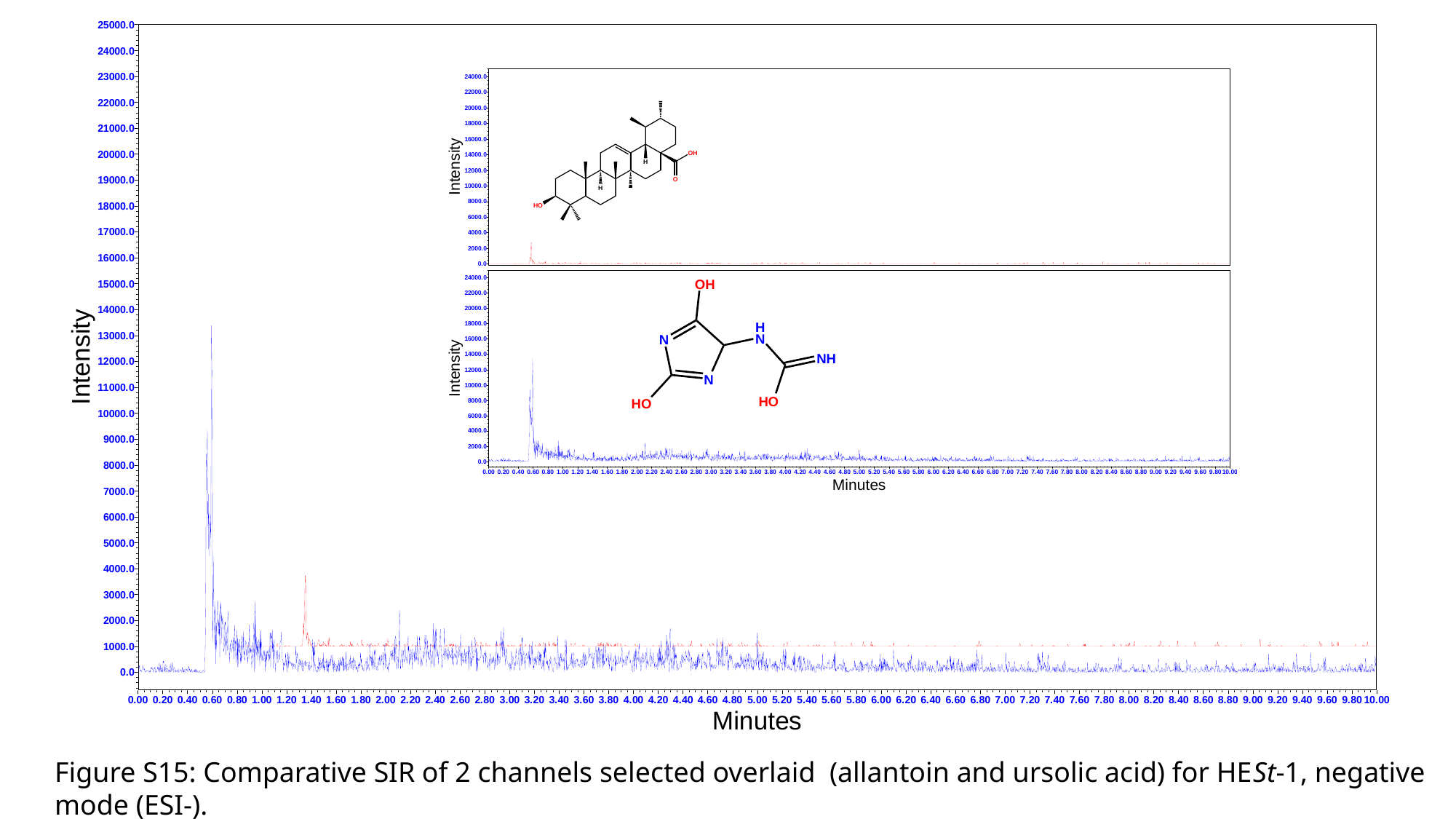

Figure S15: Comparative SIR of 2 channels selected overlaid (allantoin and ursolic acid) for HESt-1, negative mode (ESI-).

## Slide 16
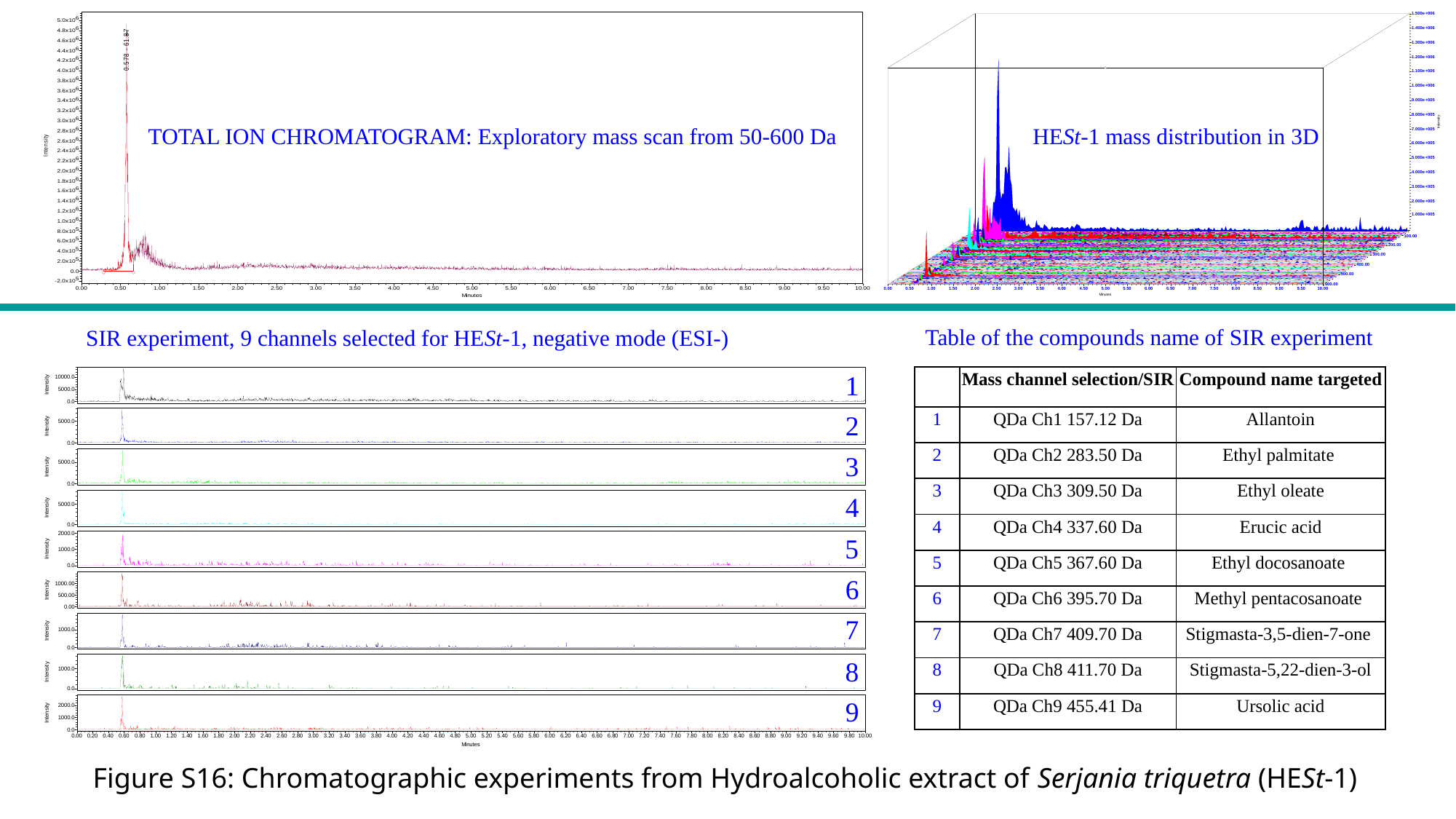

TOTAL ION CHROMATOGRAM: Exploratory mass scan from 50-600 Da
HESt-1 mass distribution in 3D
Table of the compounds name of SIR experiment
SIR experiment, 9 channels selected for HESt-1, negative mode (ESI-)
1
2
3
4
5
6
7
8
9
| | Mass channel selection/SIR | Compound name targeted |
| --- | --- | --- |
| 1 | QDa Ch1 157.12 Da | Allantoin |
| 2 | QDa Ch2 283.50 Da | Ethyl palmitate |
| 3 | QDa Ch3 309.50 Da | Ethyl oleate |
| 4 | QDa Ch4 337.60 Da | Erucic acid |
| 5 | QDa Ch5 367.60 Da | Ethyl docosanoate |
| 6 | QDa Ch6 395.70 Da | Methyl pentacosanoate |
| 7 | QDa Ch7 409.70 Da | Stigmasta-3,5-dien-7-one |
| 8 | QDa Ch8 411.70 Da | Stigmasta-5,22-dien-3-ol |
| 9 | QDa Ch9 455.41 Da | Ursolic acid |
Figure S16: Chromatographic experiments from Hydroalcoholic extract of Serjania triquetra (HESt-1)

## Slide 17
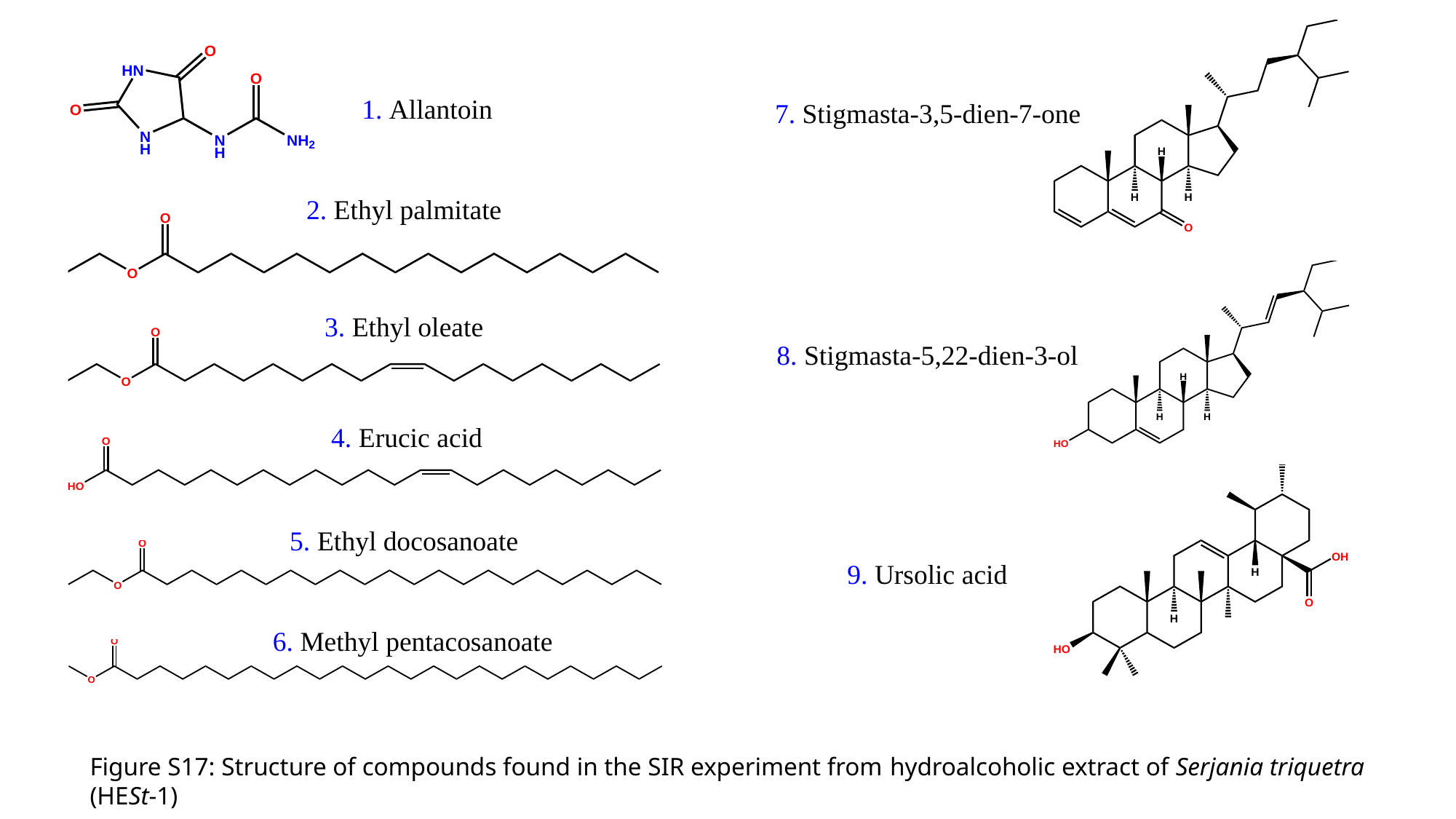

7. Stigmasta-3,5-dien-7-one
1. Allantoin
2. Ethyl palmitate
8. Stigmasta-5,22-dien-3-ol
3. Ethyl oleate
4. Erucic acid
9. Ursolic acid
5. Ethyl docosanoate
6. Methyl pentacosanoate
Figure S17: Structure of compounds found in the SIR experiment from hydroalcoholic extract of Serjania triquetra (HESt-1)

## Slide 18
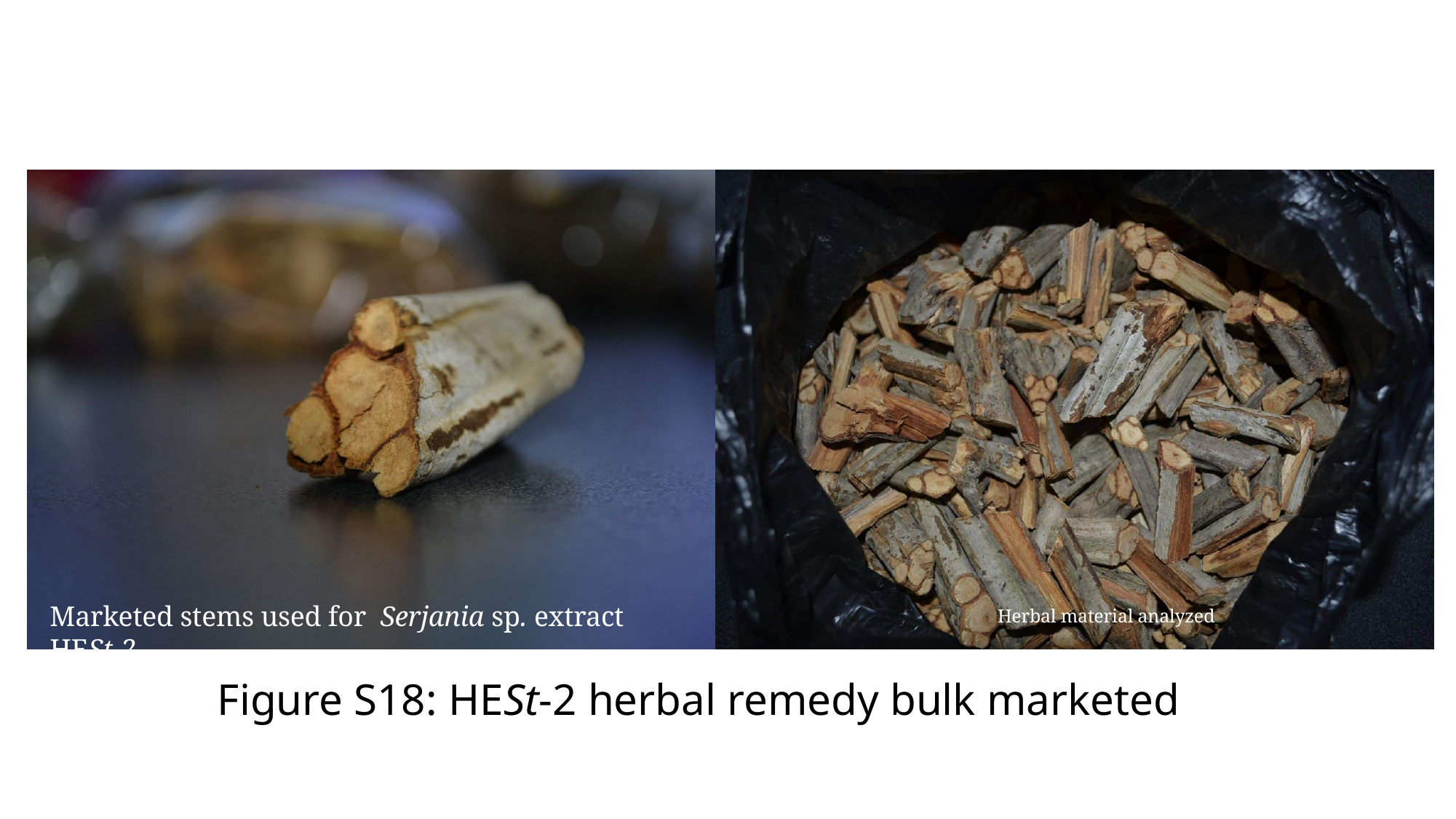

Marketed stems used for Serjania sp. extract HESt-2
Herbal material analyzed
Figure S18: HESt-2 herbal remedy bulk marketed

## Slide 19
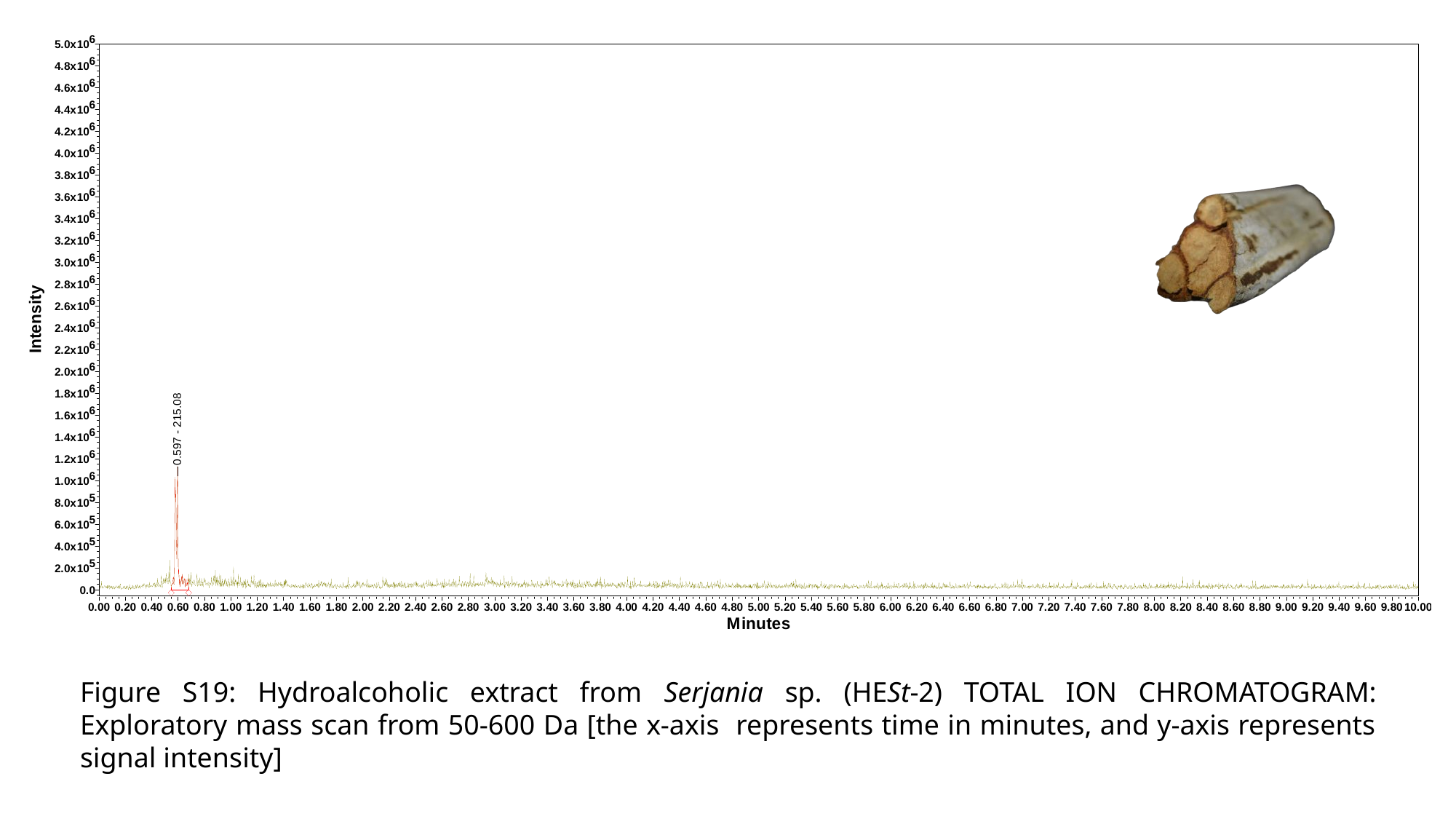

Figure S19: Hydroalcoholic extract from Serjania sp. (HESt-2) TOTAL ION CHROMATOGRAM: Exploratory mass scan from 50-600 Da [the x-axis represents time in minutes, and y-axis represents signal intensity]

## Slide 20
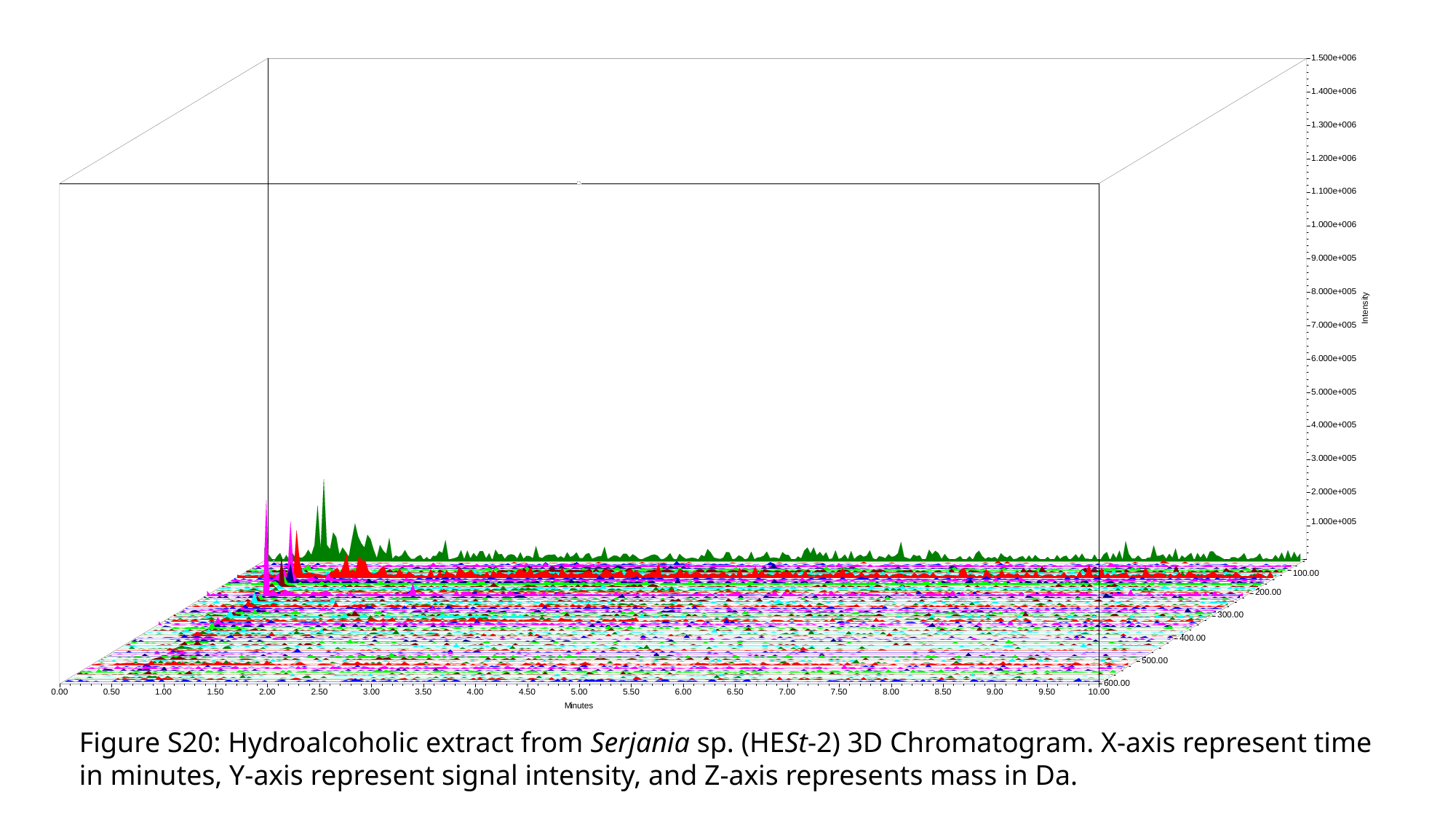

Figure S20: Hydroalcoholic extract from Serjania sp. (HESt-2) 3D Chromatogram. X-axis represent time in minutes, Y-axis represent signal intensity, and Z-axis represents mass in Da.

## Slide 21
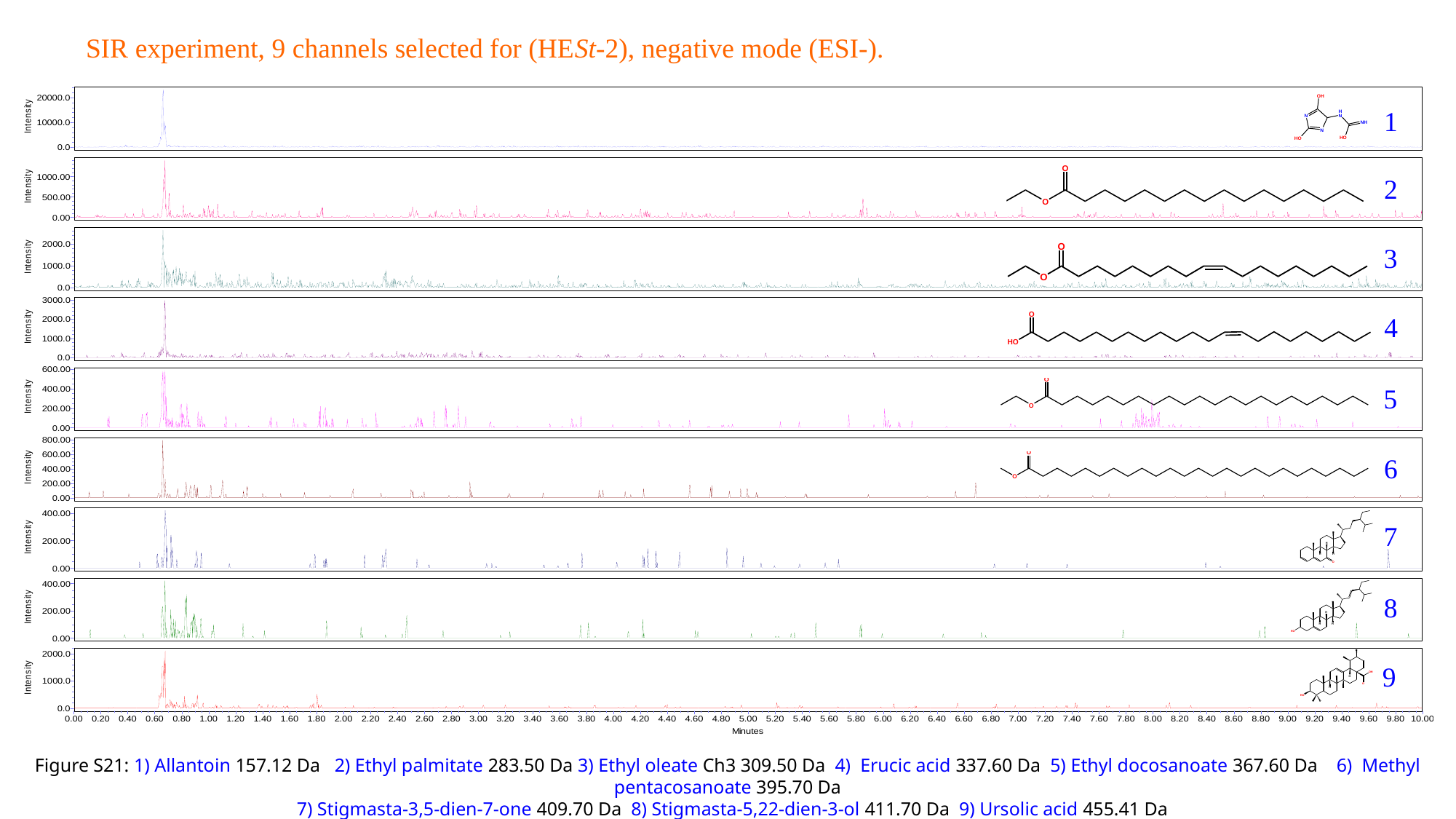

SIR experiment, 9 channels selected for (HESt-2), negative mode (ESI-).
1
2
3
4
5
6
7
8
9
Figure S21: 1) Allantoin 157.12 Da 2) Ethyl palmitate 283.50 Da 3) Ethyl oleate Ch3 309.50 Da 4) Erucic acid 337.60 Da 5) Ethyl docosanoate 367.60 Da 6) Methyl pentacosanoate 395.70 Da
 7) Stigmasta-3,5-dien-7-one 409.70 Da 8) Stigmasta-5,22-dien-3-ol 411.70 Da 9) Ursolic acid 455.41 Da

## Slide 22
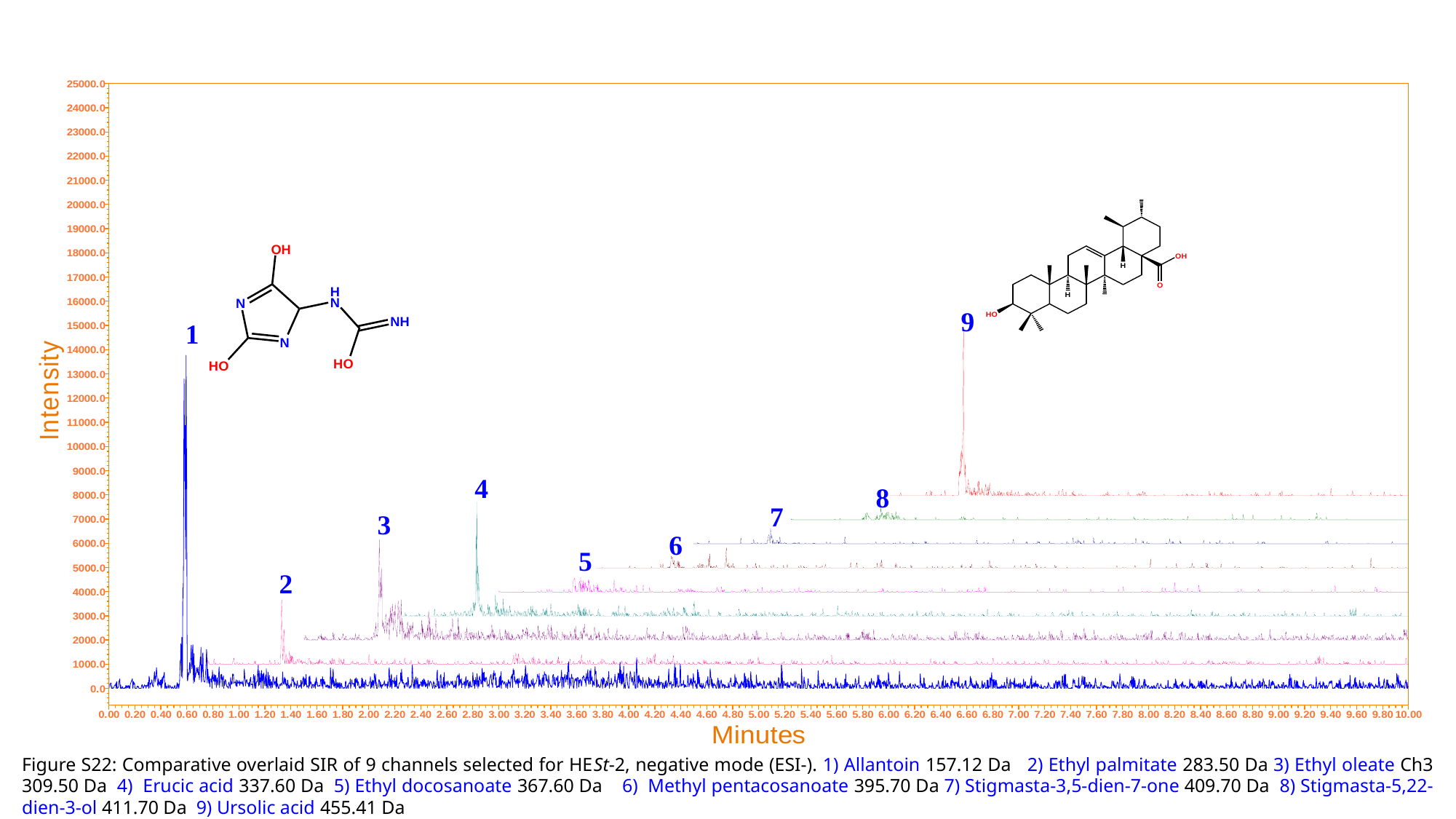

9
1
4
8
7
3
6
5
2
Figure S22: Comparative overlaid SIR of 9 channels selected for HESt-2, negative mode (ESI-). 1) Allantoin 157.12 Da 2) Ethyl palmitate 283.50 Da 3) Ethyl oleate Ch3 309.50 Da 4) Erucic acid 337.60 Da 5) Ethyl docosanoate 367.60 Da 6) Methyl pentacosanoate 395.70 Da 7) Stigmasta-3,5-dien-7-one 409.70 Da 8) Stigmasta-5,22-dien-3-ol 411.70 Da 9) Ursolic acid 455.41 Da

## Slide 23
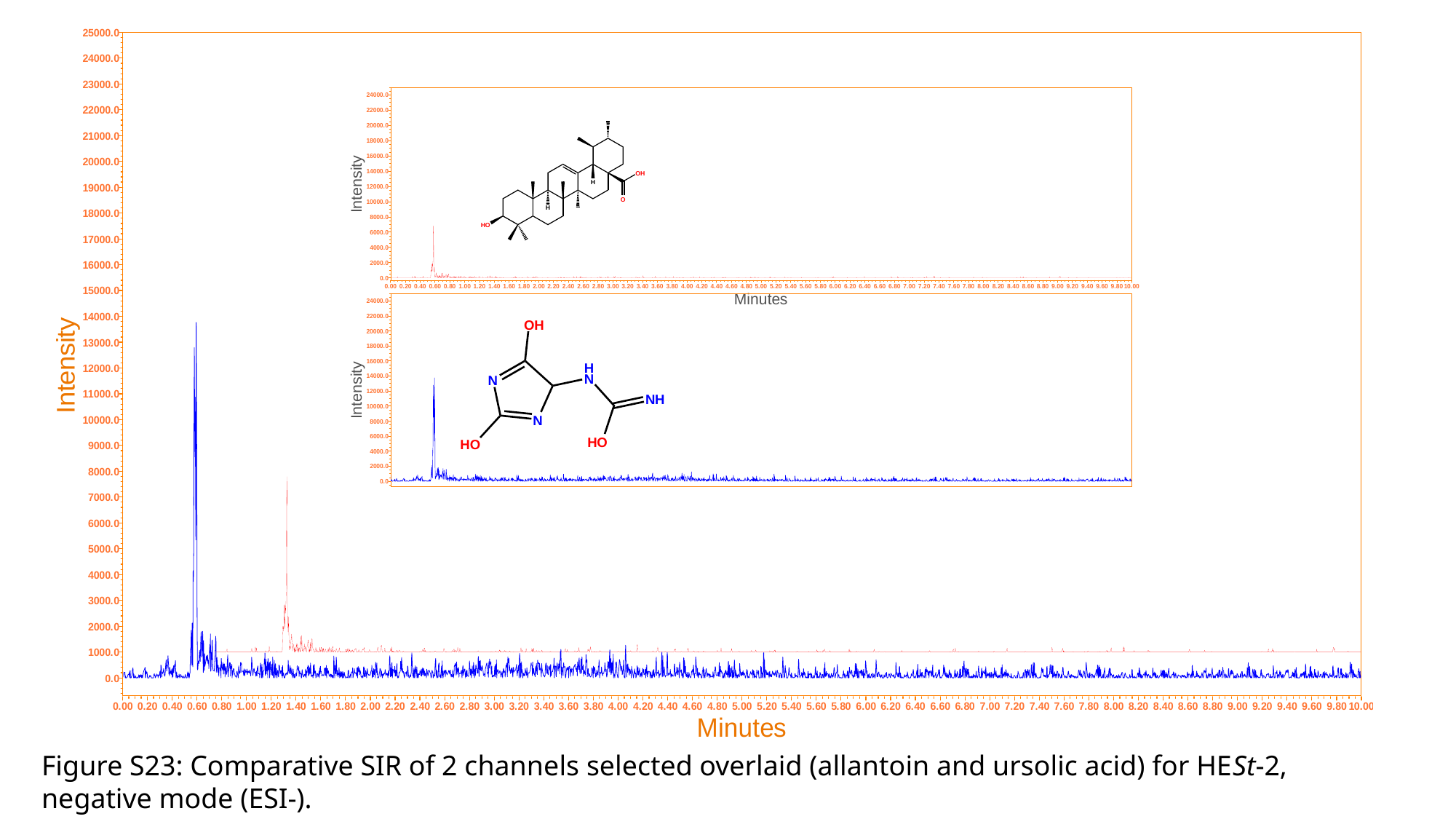

Figure S23: Comparative SIR of 2 channels selected overlaid (allantoin and ursolic acid) for HESt-2, negative mode (ESI-).

## Slide 24
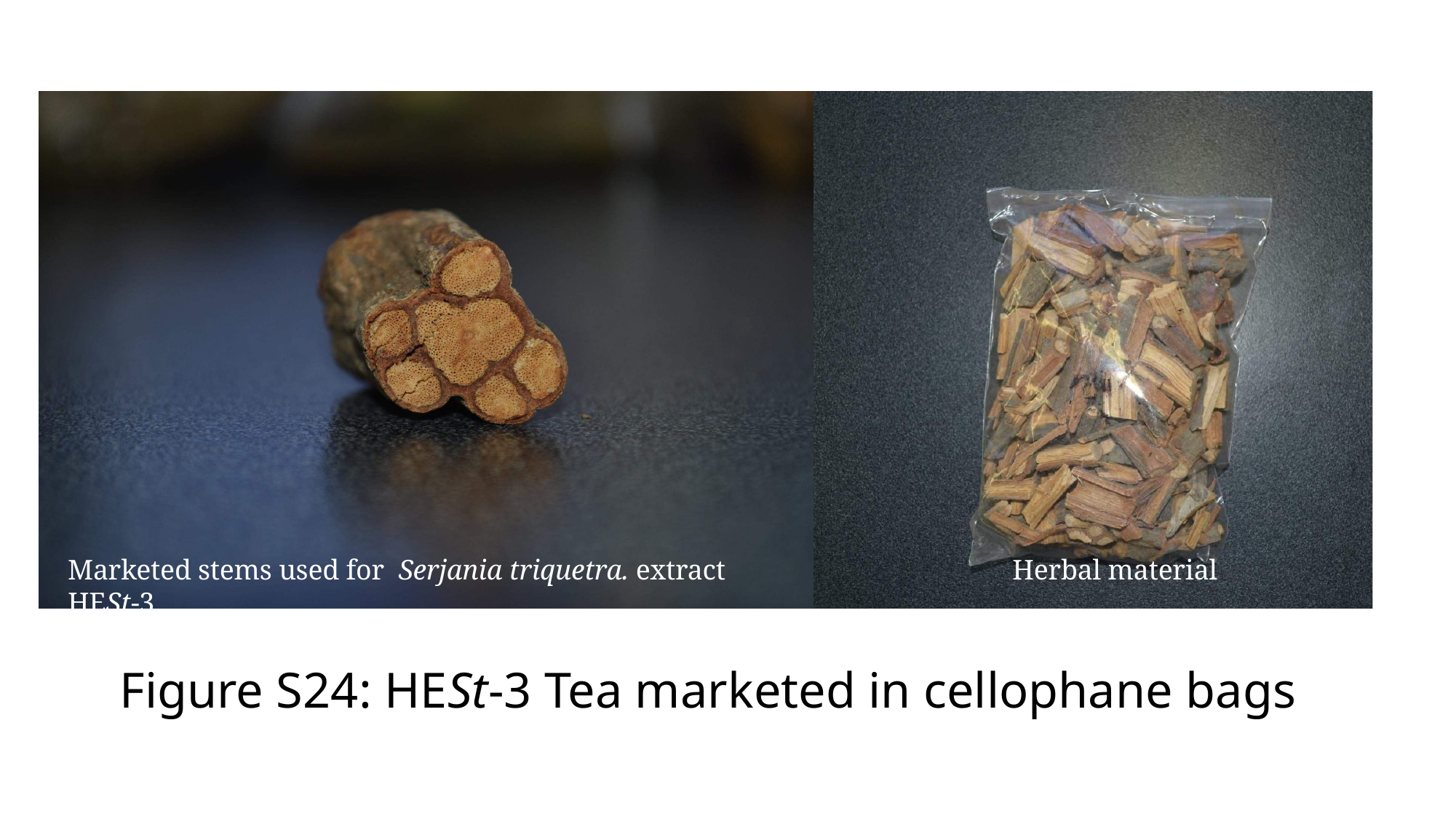

Marketed stems used for Serjania triquetra. extract HESt-3
Herbal material
Figure S24: HESt-3 Tea marketed in cellophane bags

## Slide 25
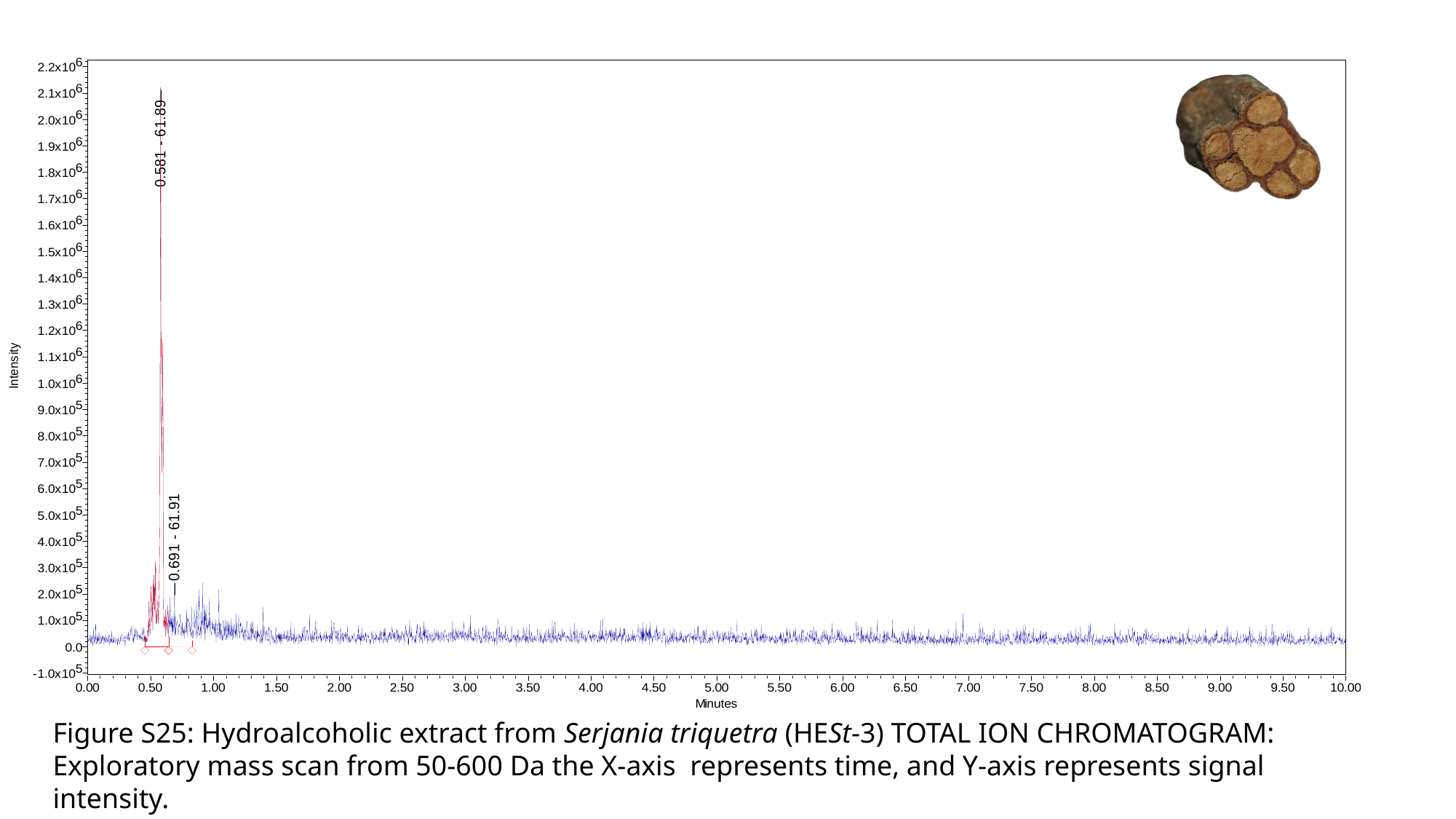

Figure S25: Hydroalcoholic extract from Serjania triquetra (HESt-3) TOTAL ION CHROMATOGRAM: Exploratory mass scan from 50-600 Da the X-axis represents time, and Y-axis represents signal intensity.

## Slide 26
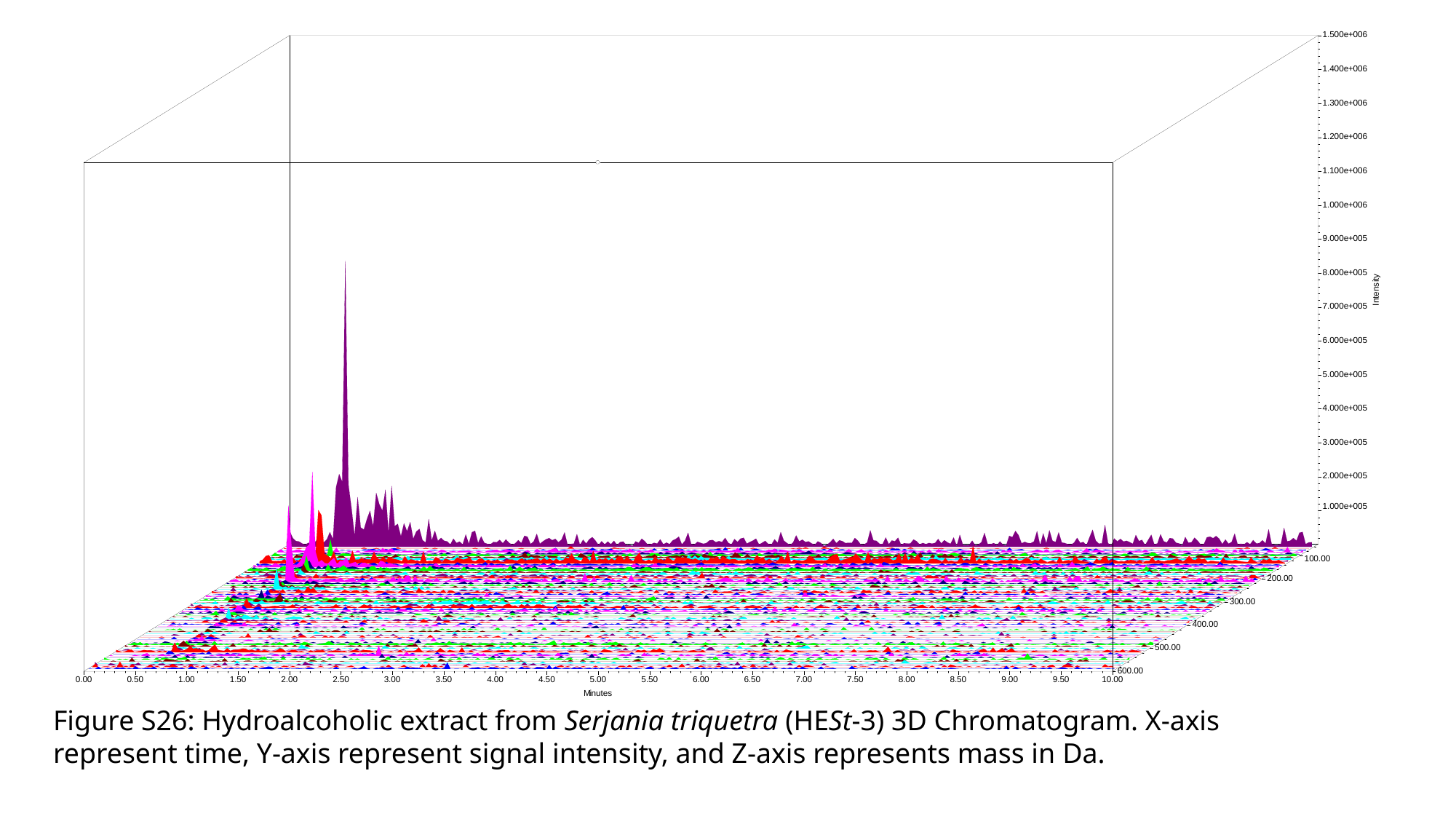

Figure S26: Hydroalcoholic extract from Serjania triquetra (HESt-3) 3D Chromatogram. X-axis represent time, Y-axis represent signal intensity, and Z-axis represents mass in Da.

## Slide 27
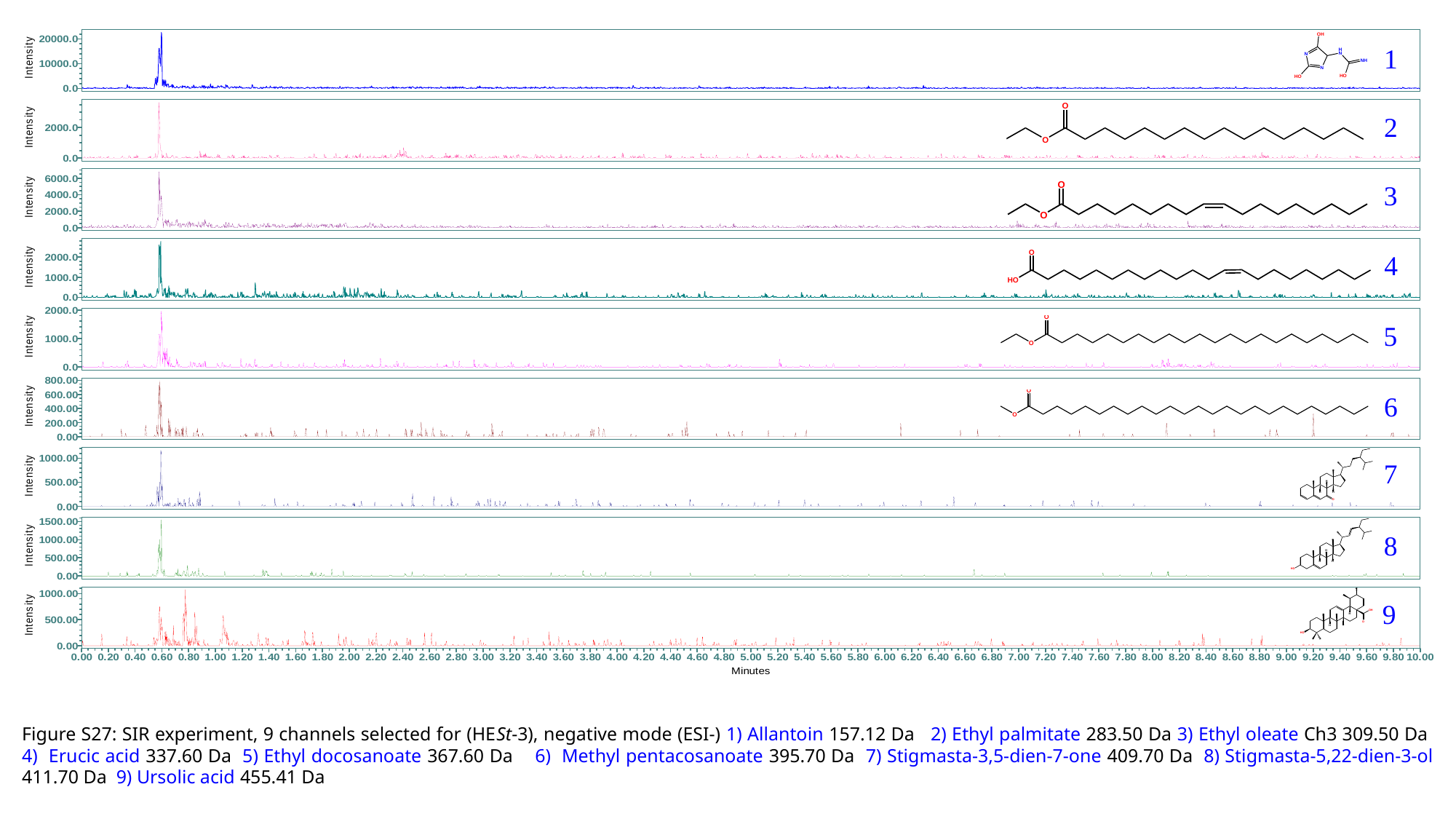

1
2
3
4
5
6
7
8
9
Figure S27: SIR experiment, 9 channels selected for (HESt-3), negative mode (ESI-) 1) Allantoin 157.12 Da 2) Ethyl palmitate 283.50 Da 3) Ethyl oleate Ch3 309.50 Da 4) Erucic acid 337.60 Da 5) Ethyl docosanoate 367.60 Da 6) Methyl pentacosanoate 395.70 Da 7) Stigmasta-3,5-dien-7-one 409.70 Da 8) Stigmasta-5,22-dien-3-ol 411.70 Da 9) Ursolic acid 455.41 Da

## Slide 28
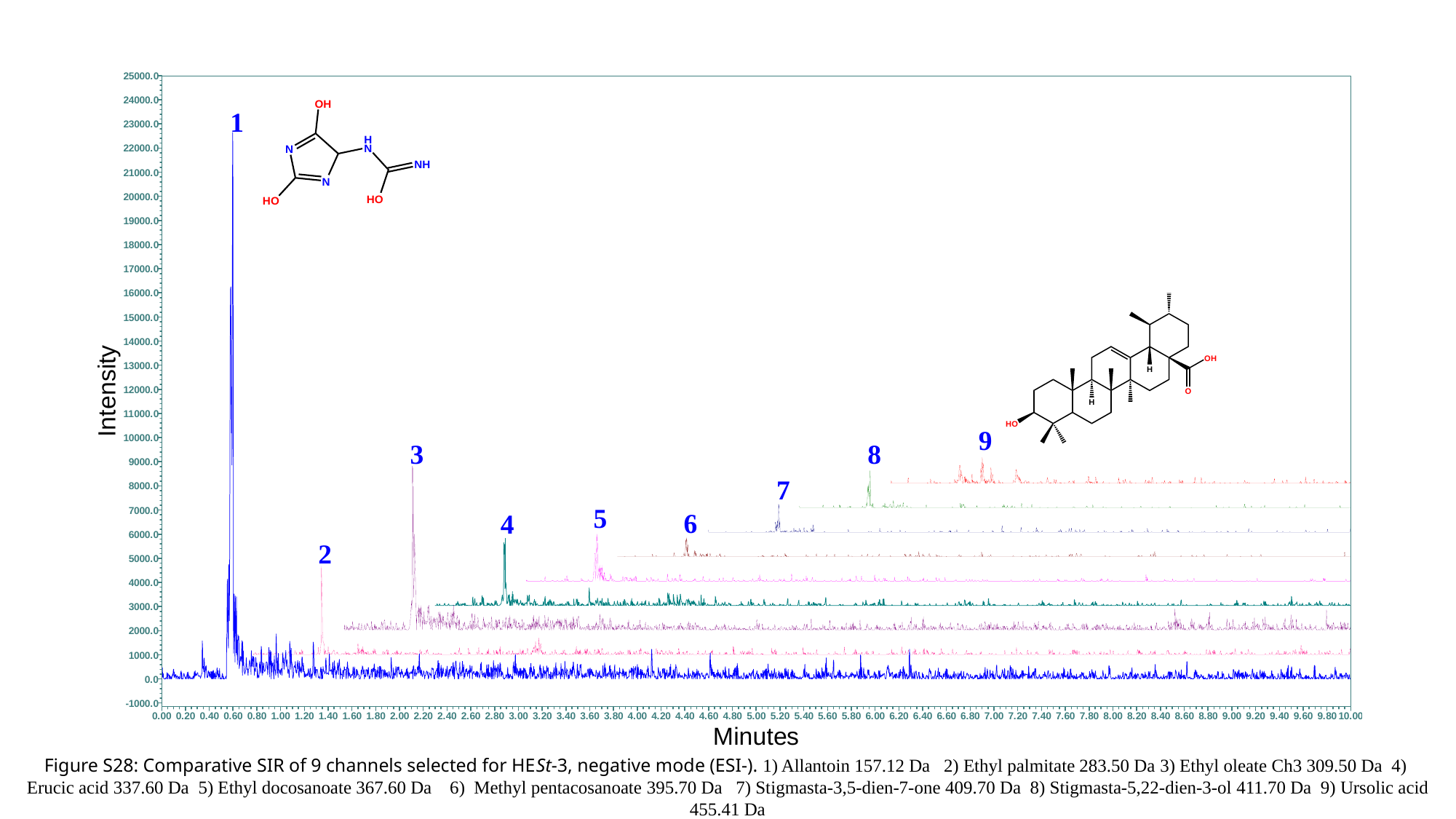

1
9
3
8
7
5
6
4
2
Figure S28: Comparative SIR of 9 channels selected for HESt-3, negative mode (ESI-). 1) Allantoin 157.12 Da 2) Ethyl palmitate 283.50 Da 3) Ethyl oleate Ch3 309.50 Da 4) Erucic acid 337.60 Da 5) Ethyl docosanoate 367.60 Da 6) Methyl pentacosanoate 395.70 Da 7) Stigmasta-3,5-dien-7-one 409.70 Da 8) Stigmasta-5,22-dien-3-ol 411.70 Da 9) Ursolic acid 455.41 Da

## Slide 29
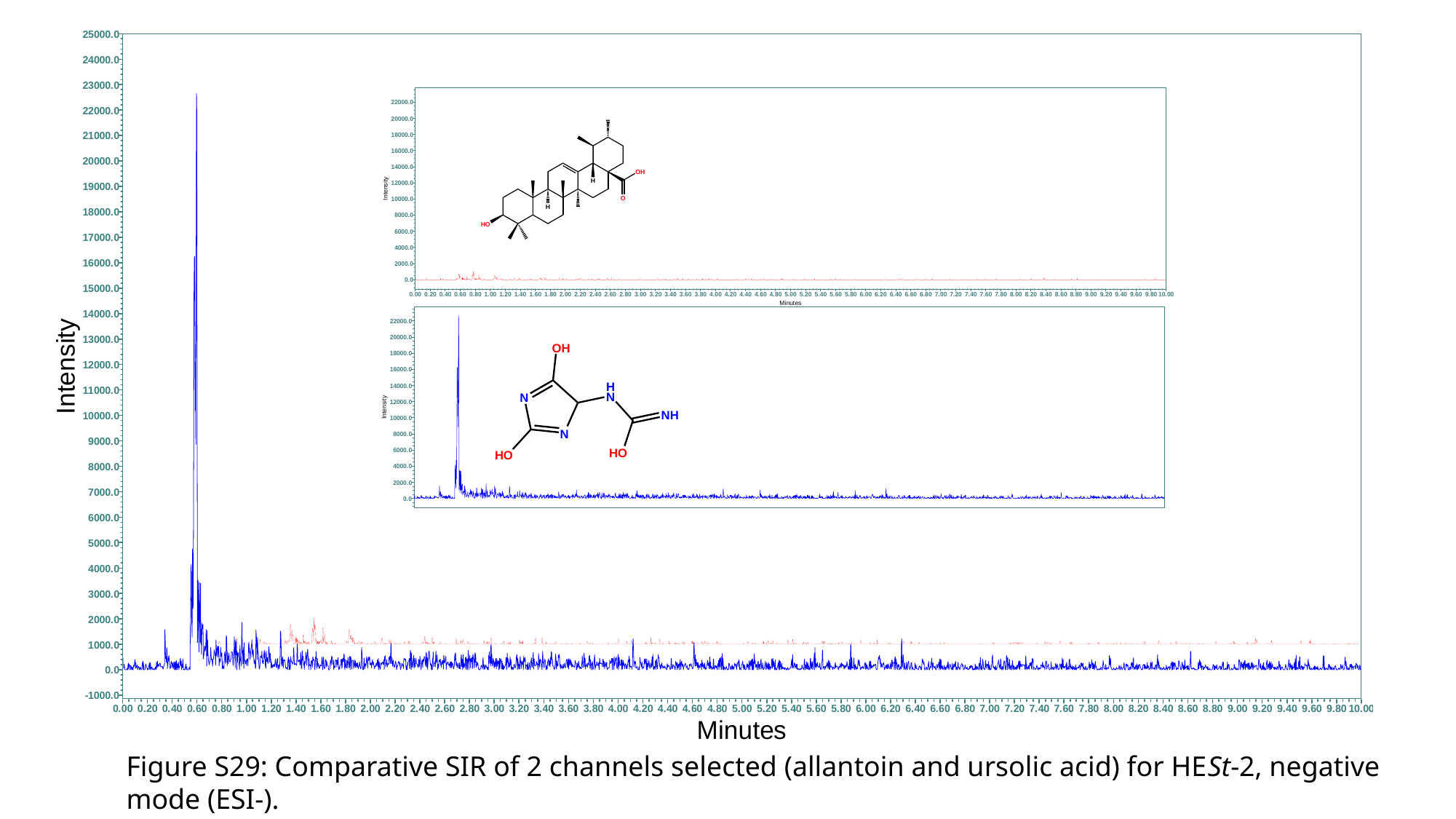

Figure S29: Comparative SIR of 2 channels selected (allantoin and ursolic acid) for HESt-2, negative mode (ESI-).

## Slide 30
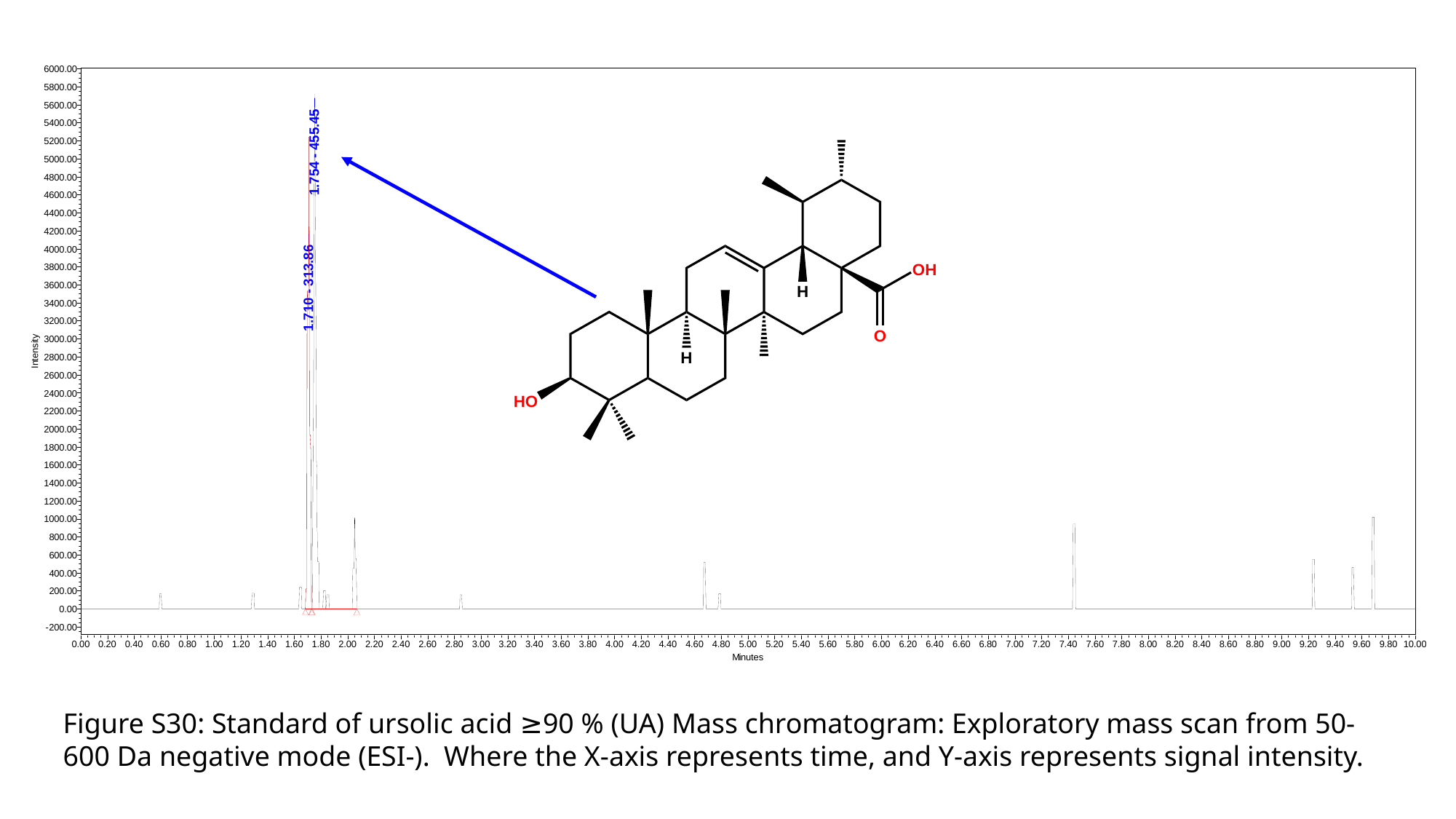

Figure S30: Standard of ursolic acid ≥90 % (UA) Mass chromatogram: Exploratory mass scan from 50-600 Da negative mode (ESI-). Where the X-axis represents time, and Y-axis represents signal intensity.

## Slide 31
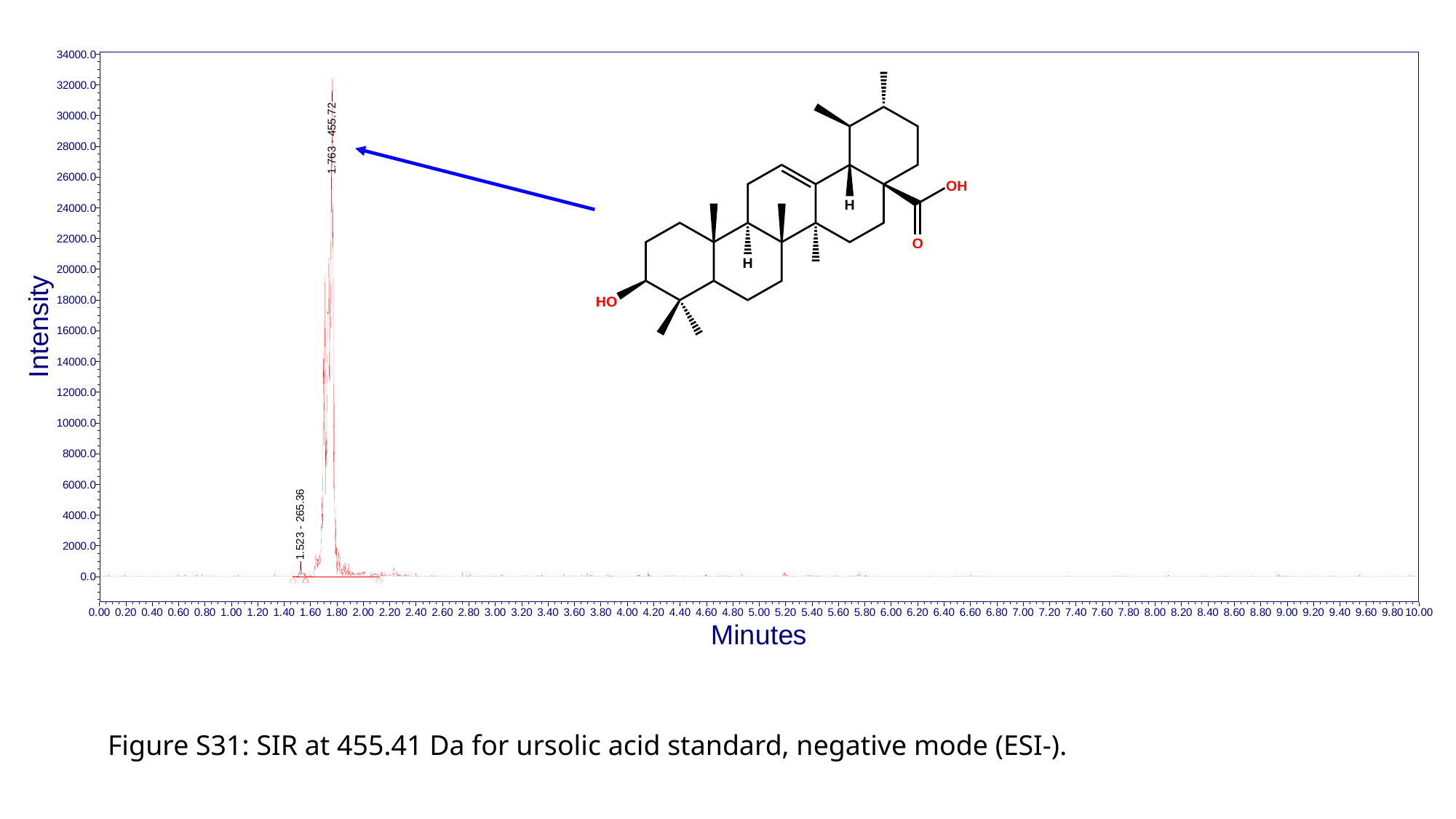

Figure S31: SIR at 455.41 Da for ursolic acid standard, negative mode (ESI-).

## Slide 32
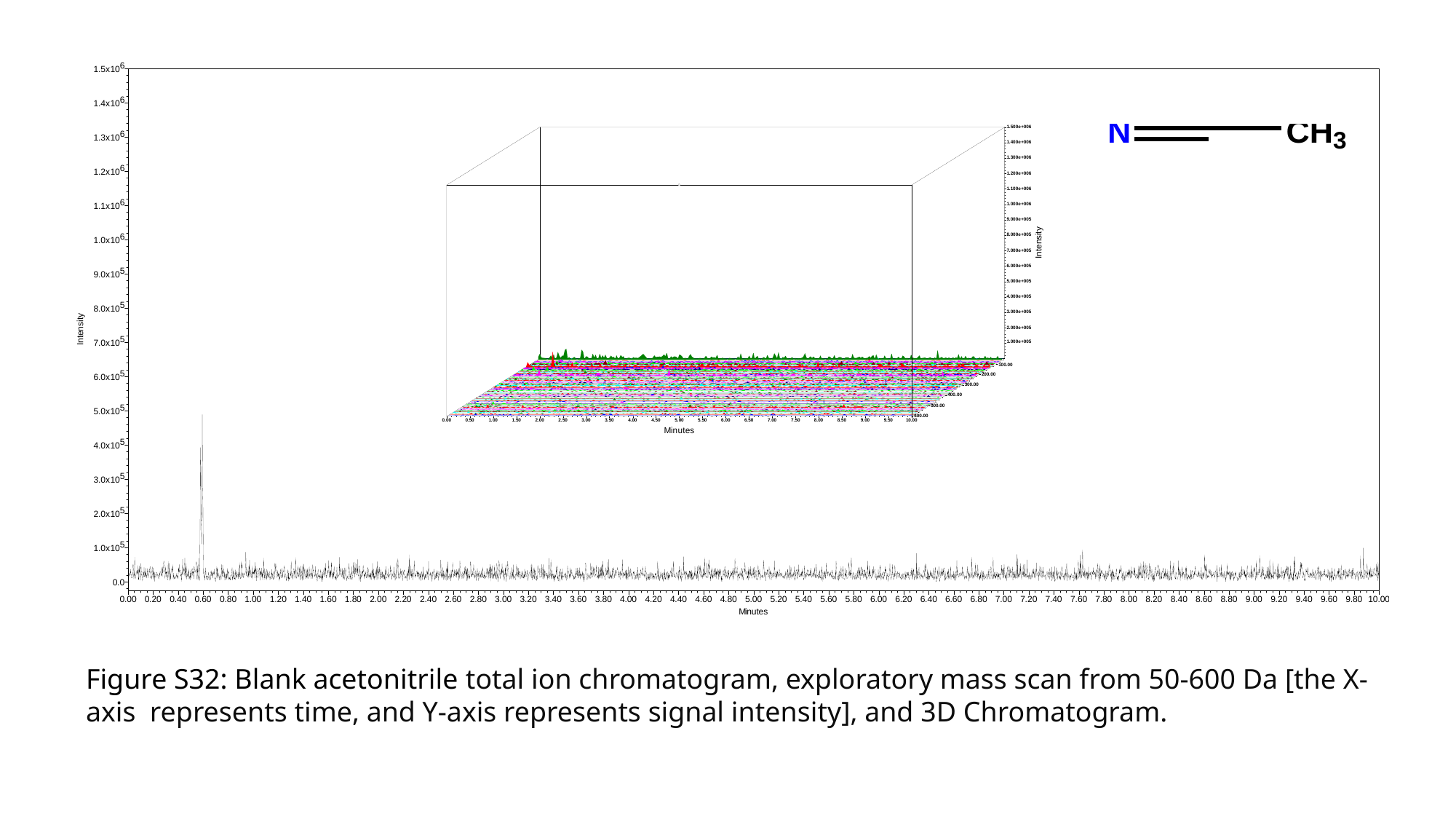

Figure S32: Blank acetonitrile total ion chromatogram, exploratory mass scan from 50-600 Da [the X-axis represents time, and Y-axis represents signal intensity], and 3D Chromatogram.

## Slide 33
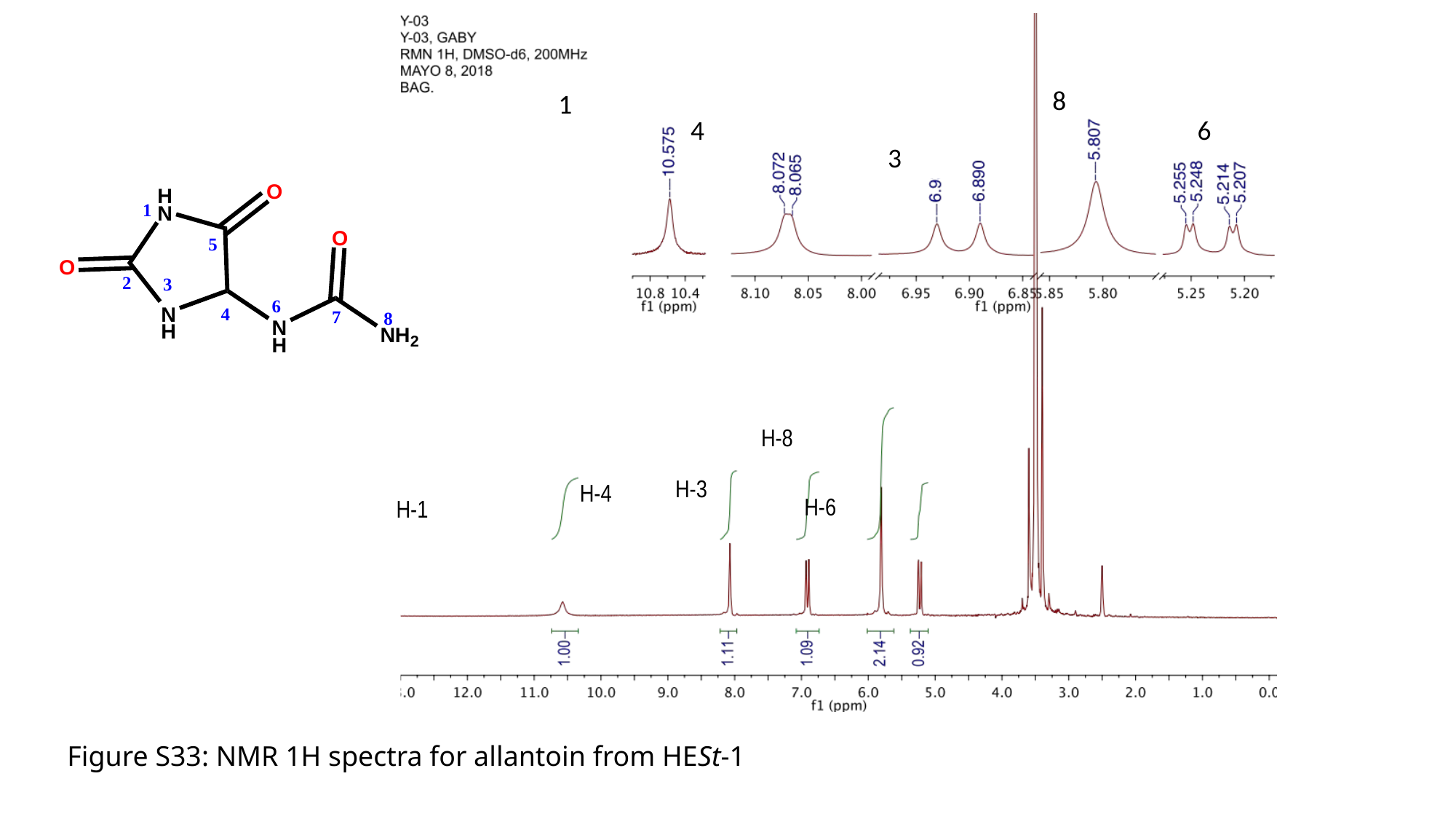

8
1
4
6
3
H-8
H-3
H-4
H-6
H-1
Figure S33: NMR 1H spectra for allantoin from HESt-1

## Slide 34
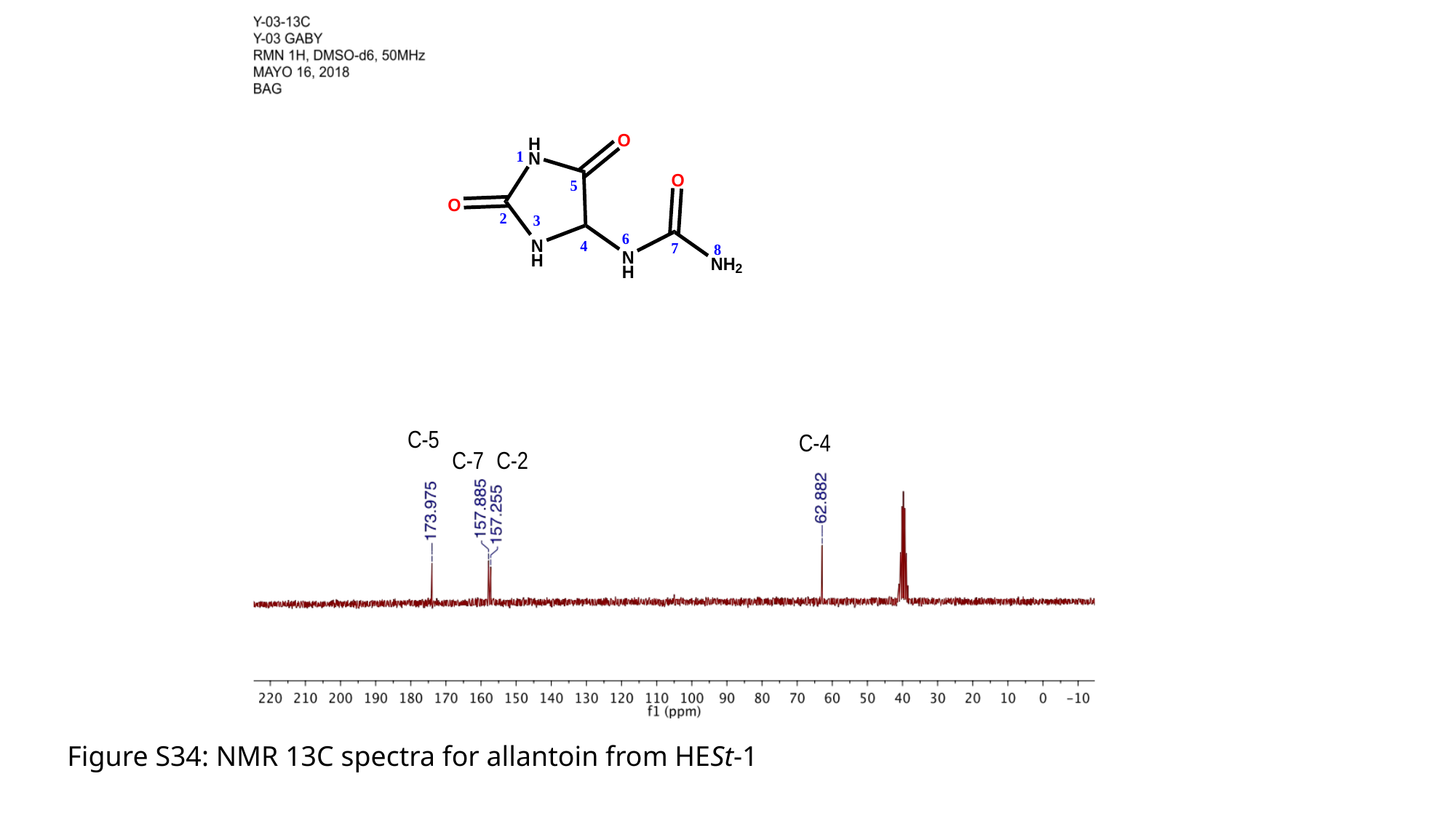

C-5
C-4
C-7
C-2
Figure S34: NMR 13C spectra for allantoin from HESt-1
